# Comparative bioinformatic and proteomic approaches to evaluate the outer membrane proteome of the fish pathogen *Yersinia ruckeri*

**DOI:** 10.1016/j.jprot.2019.02.014

**Published:** 2019-05-15

**Authors:** Michael J. Ormsby, Edward Grahame, Richard Burchmore, Robert L. Davies

**Affiliations:** a Institute of Infection, Immunity and Inflammation, College of Medical, Veterinary and Life Sciences, Sir Graeme Davies Building, University of Glasgow, Glasgow G12 8TA, UK; b Polyomics, Institute of Infection, Immunity and Inflammation, College of Medical, Veterinary and Life Sciences, TCRC, University of Glasgow, Glasgow G12 1QH, UK

**Keywords:** *Yersinia ruckeri*, Outer membrane, Proteomics, Bioinformatics

## Abstract

*Yersinia ruckeri* is the aetiological agent of enteric redmouth (ERM) disease and is responsible for significant economic losses in farmed salmonids. Enteric redmouth disease is associated primarily with rainbow trout (*Oncorhynchus mykiss,* Walbaum) but its incidence in Atlantic salmon (*Salmo salar*) is increasing. Outer membrane proteins (OMPs) of Gram-negative bacteria are located at the host-pathogen interface and play important roles in virulence. The outer membrane of *Y. ruckeri* is poorly characterised and little is known about its composition and the roles of individual OMPs in virulence. Here, we employed a bioinformatic pipeline to first predict the OMP composition of *Y. ruckeri*. Comparative proteomic approaches were subsequently used to identify those proteins expressed *in vitro* in eight representative isolates recovered from Atlantic salmon and rainbow trout. One hundred and forty-one OMPs were predicted from four *Y. ruckeri* genomes and 77 of these were identified in three or more genomes and were considered as “core” proteins. Gel-free and gel-based proteomic approaches together identified 65 OMPs in a single reference isolate and subsequent gel-free analysis identified 64 OMPs in the eight Atlantic salmon and rainbow trout isolates. Together, our gel-free and gel-based proteomic analyses identified 84 unique OMPs in *Y. ruckeri*.

**Significance:**

*Yersinia ruckeri* is an important pathogen of Atlantic salmon and rainbow trout and is of major economic significance to the aquaculture industry worldwide. Disease outbreaks are becoming more problematic in Atlantic salmon and there is an urgent need to investigate in further detail the cell-surface (outer membrane) composition of strains infecting each of these host species. Currently, the outer membrane of *Y. ruckeri* is poorly characterised and very little is known about the OMP composition of strains infecting each of these salmonid species. This study represents the most comprehensive comparative outer membrane proteomic analysis of *Y. ruckeri* to date, encompassing isolates of different biotypes, serotypes, OMP-types and hosts of origin and provides insights into the potential roles of these diverse proteins in host-pathogen interactions. The study has identified key OMPs likely to be involved in disease pathogenesis and makes a significant contribution to furthering our understanding of the cell-surface composition of this important fish pathogen that will be relevant to the development of improved vaccines and therapeutics.

## Highlights

• Most complete comparative outer membrane proteomic analysis of *Y. ruckeri* to date
• Comprised isolates of different biotypes, serotypes, OMP-types and hosts of origin
• One hundred and forty-one OMPs were predicted from four *Y. ruckeri* genomes.
• Gel-free and gel-based proteomic analyses identified 84 unique OMPs in *Y. ruckeri.*
• Key OMPs likely to be involved in disease pathogenesis identified.
• Elucidates potential roles of these diverse proteins in host-pathogen interactions.
• Furthers our understanding of the cell-surface composition of an important pathogen.

## Graphical abstract

**Figure:**
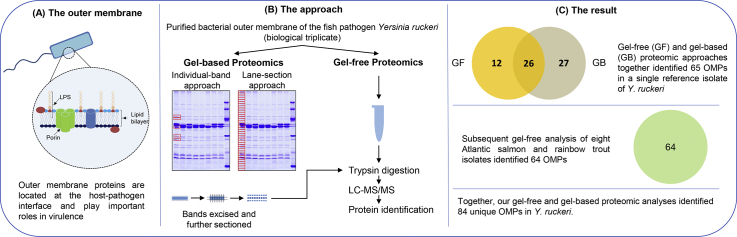

## 1 Introduction

The Gram-negative enterobacterium *Yersinia ruckeri* is the aetiological agent of enteric redmouth disease (ERM) of fish and has been recovered worldwide [[1], [2], [3], [4], [5]] from many different species [[5], [6], [7], [8], [9], [10]]. However, farmed salmonid fish, particularly rainbow trout (*Oncorhynchus mykiss,* Walbaum) and Atlantic salmon (*Salmo salar*), are most commonly affected and the disease can lead to significant economic losses [11]. In rainbow trout, ERM is characterised by a haemorrhagic septicaemia and haemorrhages in and around the oral cavity, leading to the name ‘*redmouth*’ disease [1,5,12]. The disease in Atlantic salmon, often called Yersiniosis, is characterised by a uni- or bilateral exophthalmos, with patches of haemorrhagic congestion of the iris [6,13,14]. Vaccination plays an important role in protecting rainbow trout and Atlantic salmon against *Y. ruckeri.* However, in recent years, there has been an increasing incidence of vaccine breakdown in Atlantic salmon, largely because current vaccines are aimed at rainbow trout and based on serotypes specific to this species [[15], [16], [17], [18]]. Therefore, there is a need for improved cross-protective vaccines. Extensive strain diversity has been demonstrated in *Y. ruckeri* and this is likely to have implications for vaccination strategies [[19], [20], [21], [22], [23], [24], [25], [26]]. A recent comparative study of *Y. ruckeri* recovered from Atlantic salmon and rainbow trout in Scotland demonstrated a higher level of diversity among Atlantic salmon isolates and identified the emergence of a new O-serotype [20]. In particular, Atlantic salmon isolates had outer membrane protein (OMP) profiles that were more diverse and distinct from those of rainbow trout suggesting potential roles for OMPs in host-specificity and infection. However, very little is known about the composition of the outer membrane of *Y. ruckeri* and the role of individual OMPs in virulence.

The outer membrane of Gram-negative bacteria is a highly specialised structure forming a physical and functional external barrier between the bacterial cell and its environment [27,28]. Outer membrane proteins comprise about 50% of the outer membrane mass and have a wide range of diverse functions including outer membrane biogenesis and integrity, transport, signal transduction, adherence, enzymatic activity and protection against antibiotics, detergents and toxins [[28], [29], [30], [31]]. The outer membrane is at the interface between pathogen and host, and OMPs play important roles in host-pathogen interactions including adherence and colonisation, nutrient uptake, evasion of the host immune response and tissue damage [28]. Thus, establishing the protein composition of the outer membrane of Gram-negative bacterial pathogens represents an essential step in elucidating the roles of OMPs in pathogenesis.

Bioinformatic prediction of the outer membrane proteome from genomic sequence data has been successfully used in several Gram-negative bacterial species [[32], [33], [34], [35], [36]]. Previously, we developed a bioinformatic consensus prediction pipeline which utilised ten bioinformatics programs and was used to confidently predict 98 and 107 OMPs in avian and porcine strains of *Pasteurella multocida*, respectively [37]; indeed, this approach has since been applied to several other pathogens [38,39]. A variety of gel-free and gel-based proteomic techniques have been utilised to dissect the outer membrane proteomes of different bacterial species. Gel-free proteomic approaches have been used to identify whole-cell and secreted proteins [[40], [41], [42], [43], [44], [45]] as well as proteins present in outer membrane vesicles (OMVs) [[45], [46], [47], [48]]. Subcellular fractionation of the outer membrane followed by gel-free proteomics has been successfully employed for the identification of OMPs in *Edwardsiella tarda* [49], *Coxiella burnetti* [51]*, P. multocida* [34] and *Mannheimia haemolytica* [52]. Similarly, gel-based proteomic approaches have been extensively used to characterise bacterial whole-cells, secreted proteins, OMVs and OMPs [[53], [54], [55], [56]]. Although few studies have combined both gel-free and gel-based approaches to characterise the outer membrane, coverage has generally been maximised by the use of both complementary techniques [[57], [58], [59], [60], [61], [62], [63]]. In each of these studies, bias attributed to a single methodology was compensated for by the use of the second complementary technique. Thus, the use of complementary techniques provides an improved insight into the protein composition of the bacterial outer membrane.

Very few proteomic analyses of *Y. ruckeri* have been performed*.* A shotgun proteomic analysis identified 1395 and 1447 whole-cell proteins in four isolates of *Y. ruckeri* grown under normal and iron-limiting conditions, respectively [64]. Fifty-five OMPs were identified using 2-D gel-based proteomics to compare OMP expression of immobilized *Y. ruckeri* cells with those in early and late planktonic growth although, in this case, only a single isolate was assessed [64]. The current study aimed to characterise and compare the outer membrane proteomes of eight *Y. ruckeri* isolates recovered from Atlantic salmon and rainbow trout; the outer membrane proteome was first predicted from four publicly-available genome sequences. The *Y. ruckeri* isolates were specifically selected to represent a range of biotypes, serotypes, OMP-types and virulence characteristics [66,67]. Gel-free and gel-based proteomic methods were first used to determine the outer membrane proteome of a single reference isolate and to make a comparative assessment of these two complementary approaches. Although we identified limitations, gel-free proteomics was subsequently used to identify and compare the expression of OMPs in the eight *Y. ruckeri* isolates from Atlantic salmon and rainbow trout. In this way, we aimed to identify OMPs of *Y. ruckeri* that were uniquely associated with a single host species and putatively involved in host adaptation to Atlantic salmon or rainbow trout. Such proteins could represent potential vaccine candidate antigens and form the basis for further research aimed at improving current control strategies.

## 2 Methods

### 2.1 Bioinformatic prediction of OMPs

The publicly available genomes of four isolates of *Y. ruckeri* [[68], [69], [70], [71]] were used for bioinformatic predictions (Genomes were downloaded from NCBI 07.05.2015). The properties of these isolates are summarised in Table 1. Confidently predicted OMPs were identified as described by E-Komon et al. [37] with several modifications based on programme availability. Briefly, the genomes were analysed by eight (rather than 10) prediction tools, encompassing three classes of bioinformatic predictors: (a) subcellular localisation; (b) β-barrel conformation; and (c) lipoprotein motifs. Subcellular localisation predictors included the programmes PSORTb, CELLO and SOSUI-GramN; β-barrel predictors included TMBETADISC-RBF, MCMMB and BOMP; and outer membrane lipoprotein predictors included LipoP and LIPO [37]. A consensus prediction framework was followed whereby proteins that were predicted (i) to be localised to the outer membrane by at least two subcellular localisation predictors, (ii) to have a β-barrel conformation by at least two transmembrane β-barrel predictors or (iii) to be outer membrane lipoproteins by at least one lipoprotein predictor, were considered to be putative OMPs. In several cases, two predicted proteins within a genome were determined to constitute a single functional protein and were therefore grouped as such. A list of putative OMPs within each genome was produced by integrating the results from each of the prediction categories. The putative OMPs were further scrutinised using domain, homology and literature searches to assign likely function and predict subcellular localisation with greater confidence. Based on this further information, a list of confidently predicted OMPs was generated.

**Table 1:** Properties of four *Y. ruckeri* isolates used for bioinformatic prediction of OMPs.

Strain		Host species	Phenotype		Genome				
Accession	Designation		Serotype	Biotype	Size (Mb)	GC%	Genes	Proteins	Date
PRJNA243513	ATCC29473	Rainbow trout	O1	1	3.77281	47.4	3457	3377	2014
PRJEB6967	CSF007-82	Rainbow trout	O1	1	3.83052	47.4	3483	3352	2014
PRJNA253851	37551	Atlantic salmon	O1	1	3.77549	47.6	3466	3406	2014
PRJNA237812	YRB	Rainbow trout	n/a	n/a	3.60522	47.5	3219	3079	2015

This information was gathered directly from NCBI.

### 2.2 Bacterial isolates and growth conditions

Eight representative isolates of *Y. ruckeri* recovered from infected Atlantic salmon (four) and rainbow trout (four) were selected for proteomic analyses. The properties of these isolates are presented in Table 2. Bacterial stocks, generated from a single colony, were stored at −80 °C in 50% glycerol (v/v) in tryptone soya broth (TSB; Oxoid) and were routinely subcultured on tryptone soya agar (TSA; Oxoid) at 22 °C for 48 h. Liquid cultures were prepared by inoculating three or four colonies into 10 ml volumes of TSB and incubating overnight at 22 °C with shaking at 120 rpm. For the production of OMPs, 400 μl of overnight cultures were inoculated into 400 ml volumes of TSB in 2-l Erlenmeyer flasks. These cultures were grown aerobically at 22 °C for ~16 h with shaking at 120 rpm until an OD_600nm_ of 1.0 (mid-log phase) was achieved.

**Table 2:** Properties of eight *Yersinia ruckeri* isolates included in this study.

Designation		Source			Phenotype		
Lab	Previous	Geographic origin	Host species	Year of isolation	Biotype	Serotype	OMP-type
RD6	–	U.K.	Rainbow trout	Pre 1990	2	O1	1b
RD28	BA2	U.K.	Rainbow trout	Pre 1990	1	O5	2a
RD64	F53.1/82	West Germany	Rainbow trout	1982	1	O2	2a
RD124	851,014	Denmark	Rainbow trout	1985	1	O1	3a
RD354	TW60/05	U.K.	Atlantic salmon	2005	1	O2	2a
RD366	TW90/05	U.K.	Atlantic salmon	2005	1	O5	2c
RD382	FVG 269/06	U.K	Atlantic salmon	2006	1	O1	3a
RD420	TW110/08	U.K.	Atlantic salmon	2008	1	O8	3a

### 2.3 Isolation of OMPs

**Supplementary Fig. S1:**
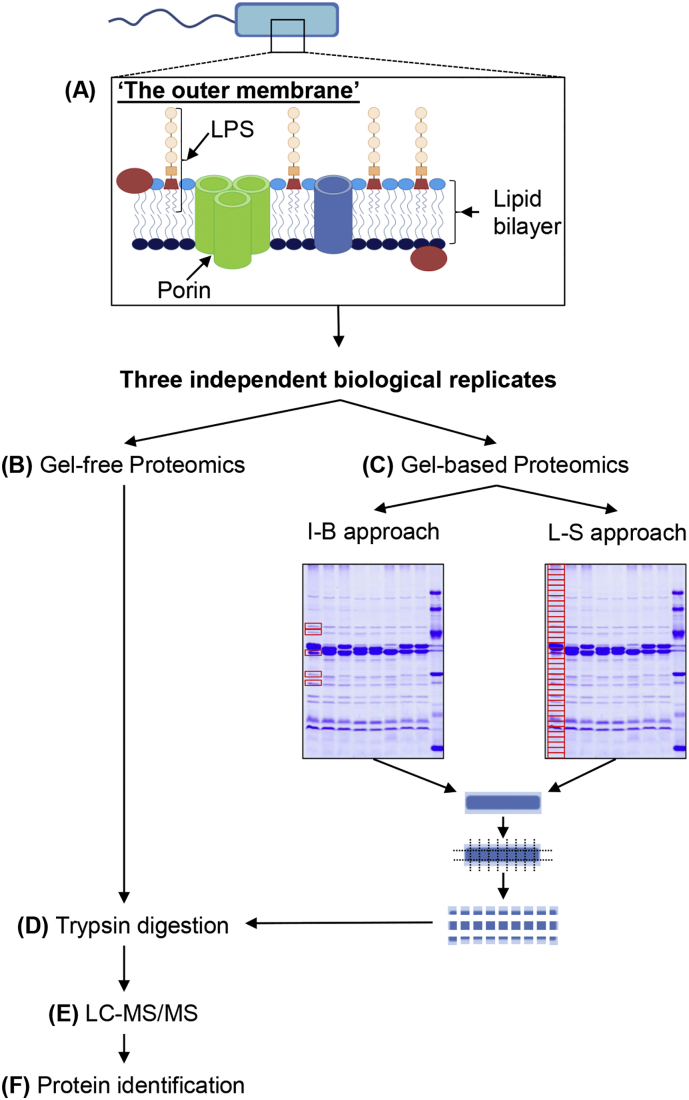
**Schematic depicting the gel-free and gel-based techniques for outer membrane protein identification.** The outer membrane was extracted and enriched by Sarkosyl extraction (A). Each independent outer membrane extract was divided into three portions and analysed by (B) gel-free and gel-based (individual band [I-B; C.1] and lane-section [L-S; C.2]) proteomic techniques. In the I-B format individual protein bands were excised, while in the L-S format the entire lane was excised. Both gel-free and gel-based samples were subjected to trypsin digestion (D), analysed by LC-MS/MS (E) and the proteins subsequently identified using MASCOT and literature searching (F).

Outer membranes of *Y. ruckeri* were isolated by Sarkosyl extraction as previously described [20]. One hundred microliter aliquots of the outer membrane suspensions were adjusted to 2 mg/ml in 20 mM Tris/HCl (pH 7.2) and the samples stored at −80 °C. Three independent biological replicates were generated and used for both gel-free and gel-based proteomic analyses (Fig. S1).

### 2.4 Gel-free proteomic analysis

Outer membrane fractions were directly digested by methanol-aided trypsin digestion as previously described [72]. Briefly, 40 μl of 2 mg/ml protein were resuspended in 88 μl of 50 mM ammonium bicarbonate and placed in an ice-cold sonicator bath for 20 min with regular vortexing at 5 min intervals, before being incubated at 60 °C for 20 min. Samples were placed on ice for 3 min before adding 120 μl of methanol and incubating for 5 min in an ice-cold sonicator bath with regular vortexing. Thirty-two microlitres of 20 μg/ml sequencing-grade trypsin (Promega) in 25 mM ammonium bicarbonate (trypsin solution) were added, followed by 120 μl of methanol. After vortexing briefly, the samples were incubated at 37 °C for 12 h. The digested samples were concentrated in an Eppendorf SpeedVac and stored at −20 °C.

### 2.5 Gel-based proteomic analysis

Outer membrane proteins were separated by 1-D SDS-PAGE as previously described [20]. Proteins were separated in either mini-gel (Bio-Rad Mini Protean II) or large-gel (Hoefer SE600) formats (10 and 20 μl of protein, respectively) and visualised by staining with Coomassie Brilliant Blue. In the case of the mini-gels, each lane was cut into 17 equally-sized gel sections which were subsequently sliced into small fragments. For the large-gels, individual bands were excised from the gel and sliced into small fragments. These two approaches will henceforth be referred to as the “lane-section” (L-S) and “individual-band” (I-B) methods, respectively. In each case, the excised and sliced gel pieces were washed three times in 100 μl of 50 mM ammonium bicarbonate in 50% (v/v) methanol and twice in 100 μl of 75% (v/v) acetonitrile before drying. The gel pieces were rehydrated with 50 μl trypsin solution and incubated at 37 °C for 12 h. Digested peptides were extracted by agitating the gel pieces in 30 μl of 5% (v/v) formic acid, followed by 30 μl 100% acetonitrile, before both solutions were pooled and transferred to a new 96-well plate and dried. Peptide samples were stored at −20 °C until analysis.

### 2.6 Nanoflow HPLC electrospray ionisation tandem MS (nLC-ESI-MS/MS)

Peptide samples prepared by both the gel-free and gel-based methods were analysed by nLC-ESI-MS/MS. The peptides were solubilised in 20 μl 2% acetonitrile with 0.1% trifluoroacetic acid and separated on a nanoflow uHPLC system (Thermo RSLCnano) before online analysis by electrospray ionisation (ESI) MS on an Amazon ion trap MS/MS (Bruker Daltonics). Peptide separation was performed on a Pepmap C18 reversed phase column (Thermo Scientific™ Acclaim™ PepMap™ 100 C18 LC Column, 3 μm particle size, 75 μm ID, 150 mm length), desalted and concentrated for 4 min on a C18 trap column followed by an acetonitrile gradient (in 0.1% [v/v] formic acid) (3.2 to 32% [v/v] for 4 to 27 min, 32 to 80% [v/v] for 27 to 36 min, held at 80% [v/v] for 36 to 41 min and re-equilibrated at 3.2%) for a total time of 45 min. A fixed solvent flow rate of 0.3 μl/min was used for the analytical column. The trap column solvent flow rate was fixed at 25 μl/min, using 2% acetonitrile with 0.1% (*v*/v) trifluoroacetic acid. MS analysis was performed using a continuous duty cycle of survey MS scan followed by up to ten MS/MS analyses of the most abundant peptides, choosing the most intense multipli-charged ions with dynamic exclusion for 120 s.

### 2.7 Data analysis

MS data were processed using Data Analyst software (Bruker) and the automated Matrix Science MASCOT Daemon server (v2.4.1). Protein identifications were assigned using the MASCOT search engine to interrogate protein sequences in the *Y. ruckeri* NCBI Genbank database, allowing a mass tolerance of 0.8 Da for both MS and MS/MS analyses. All peptides were searched against the entire NCBI *Y. ruckeri* database, which included the four genomes that had previously been used for prediction analyses. The MASCOT program assigned a probability-based MOWSE score to each protein and the identified protein deemed significant (*p* ≤ 0.05) if the corresponding MOWSE score was >18 and at least two peptides were identified.

## 3 Results

### 3.1 Bioinformatic prediction of OMPs from four *Y. ruckeri* genomes

**Fig. 1:**
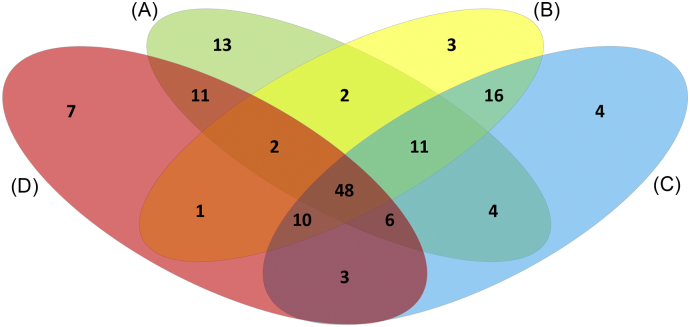
Venn diagram comparing predicted OMPs from four *Y. ruckeri* genomes. The genomes used were obtained from NCBI and represent strains ATCC 29473 (A); CSF007-82 (B); 37,551 (C); and YRB (D). Further details about these genomes are provided in Table 1.

Outer membrane proteins were predicted from four publicly available genomes of *Y. ruckeri* using eight different bioinformatic prediction programs (Table 1). Consensus bioinformatic prediction identified 97, 93, 102 and 88 confidently predicted OMPs from the rainbow trout strains ATCC29473 (encodes 3377 proteins), CSF007-82 (3352 proteins) and YRB (3079 proteins) and the Atlantic salmon strain 37551 (3406 proteins), respectively (Table S1). In total, 141 unique OMPs were confidently predicted in the four genomes. Of these, 48 (34.0%) proteins were common to all four genomes and were considered to represent the core outer membrane proteome (Fig. 1; Table S1). However, a further 29 proteins were identified in three out of the four genomes which increased the predicted core outer membrane proteome to 77 (54.6%) proteins. For future reference, the 77 proteins will be considered the core outer membrane proteome. A small number of predicted OMPs were unique to individual genomes; thus, ATCC29473, CSF007-82, YRB and 37551 contained 13 (9.2%), three (2.1%), seven (2.2%) and four (2.8%) unique proteins, respectively.

### 3.2 Comparative gel-free and gel-based outer membrane proteomic analysis of a representative *Y. ruckeri* isolate

**Fig. 2:**
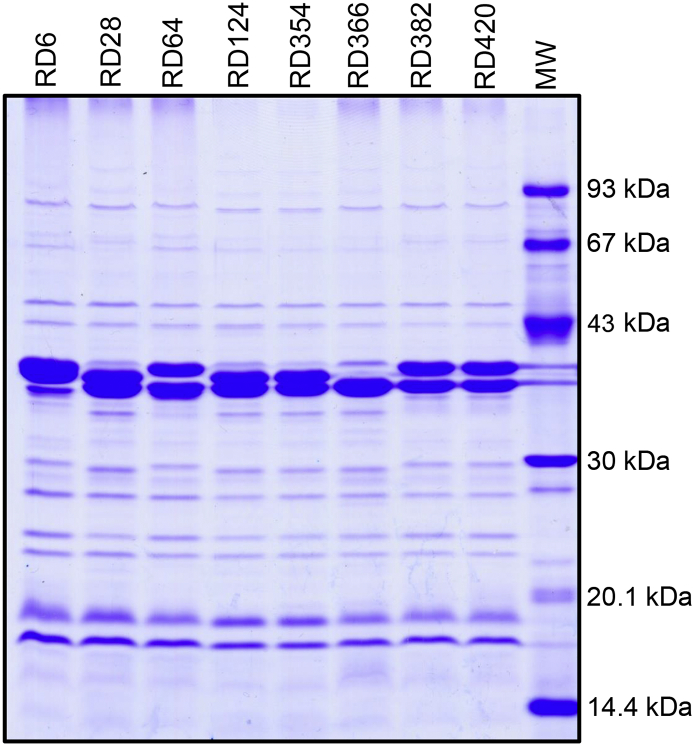
OMP-profiles of eight representative isolates of *Y. ruckeri*. Isolates RD6, RD22, RD124 and RD28 (lanes 1 to 4) were recovered from rainbow trout. Isolates RD354, RD366, RD382 and RD420 (lanes 5 to 8) were recovered from Atlantic salmon. Molecular mass standards (GE Healthcare) are shown in lane 9.

Outer membrane protein samples representative of the eight *Y. ruckeri* isolates were separated by SDS-PAGE and the OMP profiles are shown in Fig. 2. Isolate RD366 (Fig. 2) resulted in the highest number of visible protein bands, with respect to the other seven isolates, and was selected for further analysis using comparative gel-free and gel-based proteomic approaches. Outer membrane extracts of triplicate biological samples of RD366 were demonstrated to be very similar by SDS-PAGE (section 3.2.2.) and were analysed using both proteomic methodologies.

#### 3.2.1 Gel-free analysis of isolate RD366

**Fig. 3:**
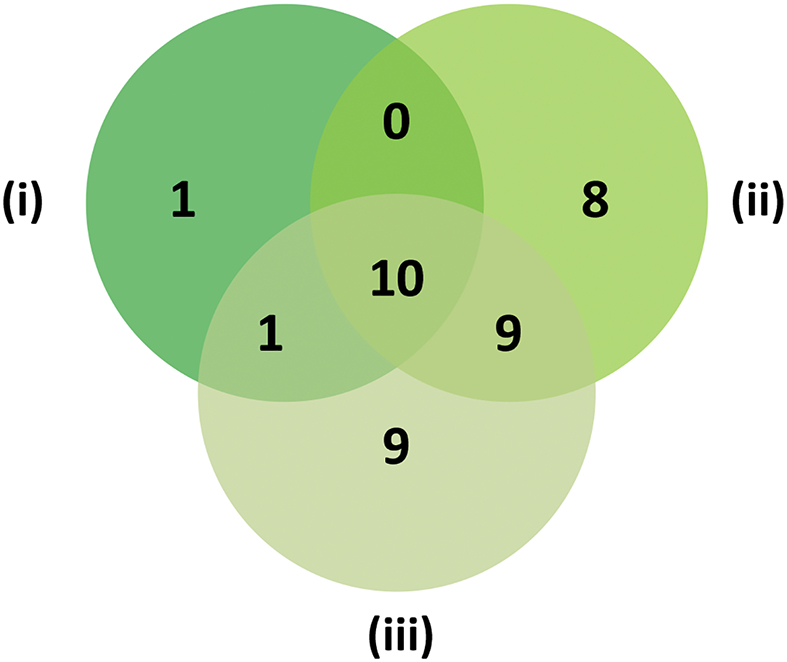
Gel-free proteomic analysis of isolate RD366. Using the gel-free proteomic approach, OMPs identified in each replicate (i to iii) are represented by a Venn diagram.

Thirty-eight OMPs were identified in the three replicate outer membrane fractions of isolate RD366 by gel-free proteomic analysis (Fig. 3; Table 3). Ten proteins were present in all three biological replicates, ten proteins occurred in two biological replicates, and 18 proteins were represented in only one biological replicate. Of the proteins identified in all three replicates, six (BamB, Lpp [Brauns], Pal, OmpA, BamA and VacJ) were associated with outer membrane biogenesis and integrity, one (OmpF) was involved in transport, two (Flagellin and FlgE) had roles in motility and one (OsmY) was categorised as having some ‘other’ function. Proteins identified in two out of three replicates included four (MetQ, ShlB/FhaC, BcsC and MltA) with enzymatic roles, one (Pcp/SlyB) involved in outer membrane biogenesis, three (MipA, YiaD/Omp16 and HslJ) with some other function and two (YeeJ and TcyP) of unknown function. Proteins identified in only a single replicate included four (BamC, Slp family, BamD and LptE/RlpB) involved in outer membrane biogenesis and integrity, seven (OmpC.1, OmpE, TolC, HemR, BtuB, OprC and OmpC.2) with roles in transport, one (FliD) involved in motility, two (YfhG/QseG and YraP) with some other function, three (YqjD, C-terminal protease and RupA) of unknown function and one hypothetical protein (hypothetical protein 5).

**Table 3:**
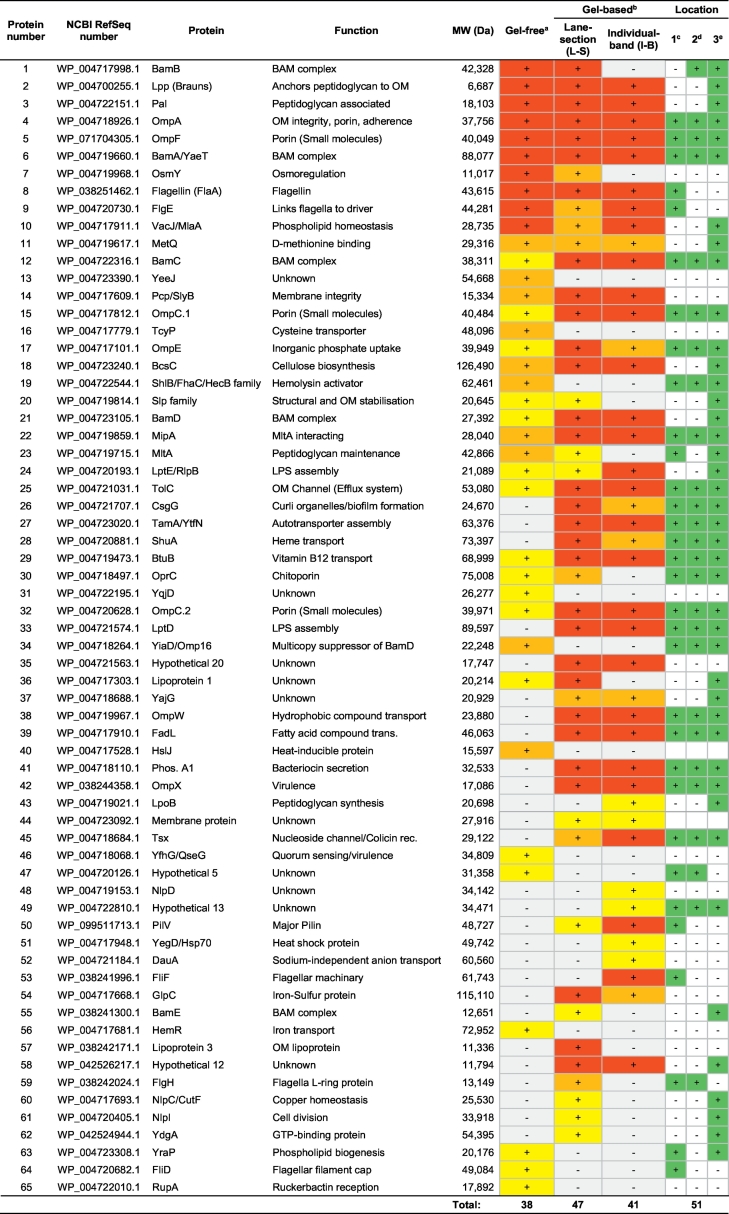
Comparison of proteins identified in isolate RD366 using gel-based and gel-free proteomic methods.

^a^Colourations indicate proteins identified in three replicates (red); two replicates (orange); and one replicate (yellow), respectively. ^b^Green colouration in prediction column is purely for visualisation. ^c^Proteins identified by subcellular localisation prediction programmes; ^d^Proteins identified by β-barrel prediction programmes; ^e^Proteins identified by lipoprotein prediction programmes.

#### 3.2.2 Gel-based analysis of isolate RD366

**Fig. 4:**
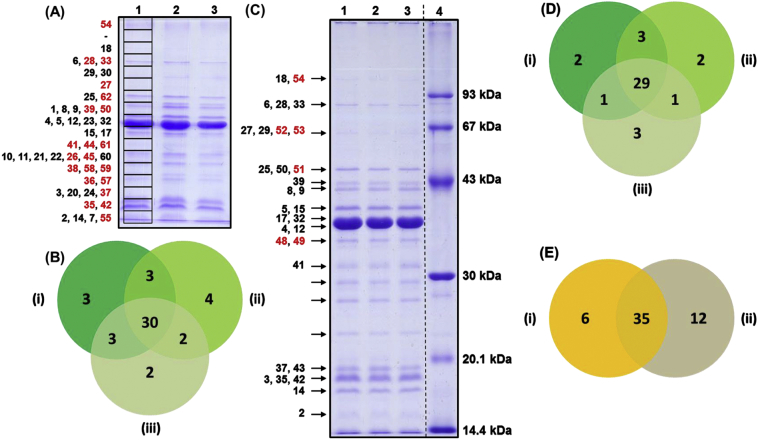
Gel-based proteomic analysis of isolate RD366. Using the L-S approach (A), segments of the gel were excised as indicated by the boxes in lane 1. Each lane was divided into 17 sections (~3 mm deep). Identified OMPs are labelled numerically and correspond to those in Table 3, column 1. OMPs identified in each replicate (i to iii) are represented by a Venn diagram (B). Using the I-B approach (C), specific OMPs were excised as indicated by arrows. Identified OMPs are labelled numerically and correspond to those in Table 3, column 1. OMPs identified in each replicate (i to iii) are represented by a Venn diagram (D). OMPs identified by both I-B (i) and L-S (ii) approaches are summarised in (E). Proteins labelled in red (A & C) were uniquely identified using gel-based methods. (For interpretation of the references to colour in this figure legend, the reader is referred to the web version of this article.)

Forty-seven proteins were identified in the three replicate outer membrane fractions of isolate RD366 using the L-S gel-based approach (Fig. 4A; Table 3). The reproducibility of the method is highlighted in Fig. 4B. Of these 47 proteins, 30 occurred in all three replicates: nine were associated with outer membrane biogenesis and integrity (BamB, Lpp [Brauns], Pal, OmpA, BamA/YaeT, BamC, Pcp/SlyB, BamD and LptD); nine had roles in transport (OmpF, OmpC.1, OmpE, TolC, ShuA, BtuB, OmpC.2, OmpW and FadL); two proteins were involved in adherence (CsgG and TamA/YtfN); single proteins had functions in motility (Flagellin [FlaA]) and enzymatic activity (BcsC); two proteins had hypothetical functions (Hypothetical 20 and Hypothetical 12); four had other functions (MipA, Phos. A1, OmpX and GlpC); and two were of unknown function (Lipoproteins 1 and 3). Thirty-eight proteins were present in at least two replicates, which in addition to those present in all three replicates, included one protein involved in outer membrane biogenesis (VacJ/MlaA), three involved in transport (OprC, Tsx and OsmY), two with roles in motility (FlgH and FlgE), one of unknown function (YajG) and one of some other function (MetQ). Nine proteins (Slp family, MltA, LptE/RlpB, membrane protein, PilV, BamE, NlpC, NlpI, and YdgA) were identified only in single replicates.

Forty-one proteins were identified by excision of all visible bands of isolate RD366 using the I-B gel-based approach (Fig. 4C; Table 3). The reproducibility of this method is highlighted in Fig. 4D. Of the 41 proteins identified, 29 proteins were present in all three replicates: ten were associated with outer membrane biogenesis and integrity (Lpp [Brauns], Pal, OmpA, BamA/YaeT, VacJ/MlaA, BamC, Pcp/SlyB, BamD, LptE/RlpB and LptD); eight had roles in transport (OmpF, OmpC.1, TolC, BtuB, OmpC.2, OmpW, FadL and Tsx); two were involved in adherence (TamA/YtfN and PilV); three proteins had functions in motility (Flagellin [FlaA], FlgE and FliF); one had enzymatic activity (BcsC); two proteins had hypothetical functions (Hypothetical proteins 12 and 20); and three had other functions (MipA, Phos. A1 and OmpX). Thirty-five proteins were present in at least two replicates which, in addition to those present in all three replicates, included two proteins with transport and receptor activities (OmpE and ShuA), one with a role in adherence (CsgG), one of other function (MetQ) and one of unknown function (YajG). Seven proteins (MetQ, LpoB, membrane protein, NlpD, hypothetical protein 13, YegD/Hsp70 and DuaA) were identified only in single replicates.

Combining the proteins identified in isolate RD366 by both the L-S and I-B approaches resulted in the identification of 53 unique OMPs, of which 46 (86.8%) had been predicted (Fig. 4E; Table 3). Thirty-five OMPs were identified using both gel formats, twenty-three of which were present in all replicates; six proteins (YegD/Hsp70, FliF, hypothetical protein 13, LpoB, NlpD and DuaA) were identified exclusively by the I-B approach; and 12 proteins (BamB, OsmY, Slp family protein, MltA, OprC, Lipoproteins 1 and 3, BamE, FlgH, NlpC/CutF, NlpI and YdgA) were identified only by the L-S approach. Notably, the 12 proteins identified exclusively using the L-S approach corresponded with regions of the gel that contained no visible bands and it is unlikely that these would have been identified by excision of visible bands only.

#### 3.2.3 Comparative gel-free and gel-based analyses of isolate RD366

**Fig. 5:**
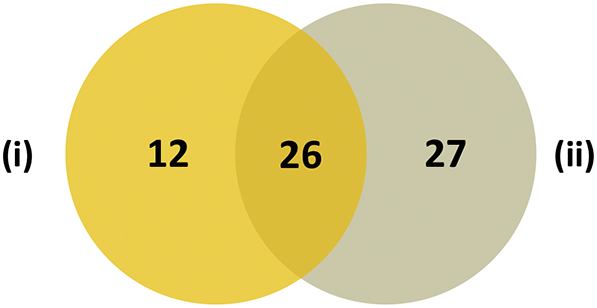
Comparison of gel-free and gel-based proteomic analyses of isolate RD366. Twenty-six proteins were identified in isolate RD366 by both gel-free and gel-based proteomic analyses. Twelve proteins were uniquely identified by gel-free analysis (i) and 27 proteins were uniquely identified by gel-based analysis (ii). The comparative list of proteins is presented in Table 3.

Sixty-five OMPs were identified in isolate RD366 using a combination of the gel-free and gel-based approaches (Table 3). Twenty-six proteins were identified using both methods, 12 proteins were identified exclusively by the gel-free approach and 27 OMPs were identified only using the gel-based approach (Fig. 5).

Of the 12 proteins identified solely by gel-free proteomics, none were identified in all three replicates; the proteins YeeJ, TcyP, ShlB/FhaC/HecB, YiaD/Omp16 and HslJ were identified in two replicates and the remaining seven proteins were identified in only a single replicate.

Of the 27 proteins identified only by the gel-based approaches (L-S and I-B), eight proteins were identified in all three replicates by both methods; these included the proteins TamA/YtfN, LptD, OmpW, FadL, Phospholipase A1, OmpX and the hypothetical proteins 12 and 20. Seven proteins were identified in at least one replicate of L-S and at least one replicate of I-B methods; these included CsgG, ShuA, YajG, Membrane protein, Tsx, PilV and GlpC. The remaining 12 proteins were identified only by a single method - six by L-S and six by I-B. Of these, only the proteins FliF, Lipoprotein 3 and FlgH were identified in at least two replicates (FliF - three replicates, I-B; Lipoprotein 3 - two replicates, L-S; FlgH - two replicates, L-S). Comparison of gel-free and gel-based methodologies suggestS that the proteomic method employed did not confer an advantage to the identification of any individual protein type. However, gel-based proteomics enabled the identification of a greater number of individual proteins than the gel-free approach.

### 3.3 Outer membrane proteomic analyses of eight representative *Y. ruckeri* isolates

**Supplementary Fig. S2:**
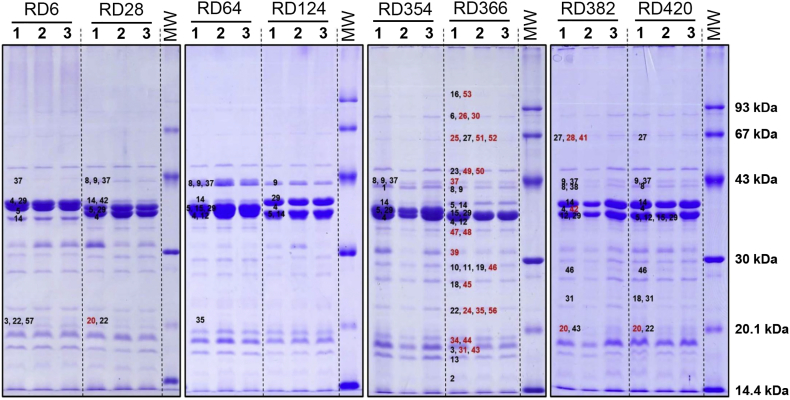
**The excised bands from OMP profiles of representative isolates of Y. ruckeri separated by 1D SDS-PAGE.** (A) Represents isolates RD6 (lanes 1-3) and RD28 (lanes 4-6); (B) represents isolates RD64 (lanes 1-3) and RD124 (lanes 4-6); (C) represents isolate RD354 (lanes 1-3) and reference isolate RD366 (lanes 4-6); (D) represents isolates RD382 (lanes 1-3) and RD420 (lanes 4-6). Lane 7 of each panel represents a MW standard (GE Healthcare, UK). Identified proteins are labelled numerically and correspond to Tables S-3 and S-4. Numbers labelled in red indicate proteins that were uniquely identified using gel based methods.

The outer membrane proteomes of the eight *Y. ruckeri* isolates were subsequently analysed. Triplicate outer membrane fractions of each isolate were generated and the uniformity of the SDS-PAGE profiles confirmed (Fig. S2). Recognising the limitations, gel-free proteomics was used to analyse these three biological replicates on the basis of cost-effectiveness. Subsequently, OMP samples were separated on large-format gels for enhanced resolution, and selected protein bands were excised and analysed to identify those proteins that exhibited visible variation (both in terms of molecular mass and degree of expression) in the isolates with respect to both RD366 and each other (Fig. S2).

#### 3.3.1 Gel-free analysis

A total of 64 unique OMPs were identified by gel-free analysis in the eight *Y. ruckeri* strains and these ranged from 33 proteins in isolate RD382 to 46 proteins in isolate RD6 (Table S2). Seven OMPs (BamB, Lpp [Brauns], Pal, OmpA, OmpF, BamA/YaeT and OsmY) were identified in all three replicates of all eight isolates (85% of these represented core proteins [Table S1]). Flagellin (FlaA) and the flagellar apparatus protein (FlgE) were identified in all isolates (in three replicates in most isolates) with the exception of RD6 (flagellin [FlaA] was considered a core protein [Table S1]). Notably, RD6 is a non-motile, biotype 2 isolate whereas all the other isolates are motile, biotype 1. Outer membrane proteins identified in two or three replicates of each isolate increased the number of proteins to 12 (Table S2). These included six proteins (BamB, Lpp [Brauns], Pal, OmpA, BamA/YaeT and MltA) involved in OM biogenesis and integrity, single proteins involved in transport (OmpF), enzymatic activity (MetQ) and adherence (ShlB/FhaC/HecB family protein), and three proteins with other functions (OsmY, YeeJ and YiaD/Omp16) (Table S2). When proteins that were identified in at least one replicate of every isolate were considered the number of proteins increased to 21 (and included also VacJ/MlaA, TcyP, BamC, Pcp/SlyB, OmpE, BcsC, HslJ, TolC and YfhG/QseG).

The majority of these 21 proteins had housekeeping functions but several OMPs were identified which had potential roles in virulence. These included proteins involved in adhesion such as PqiB/MAM7, ShlA/FhaA/HecA family adhesin/hemolysin, filamentous hemagglutinin and CsgG. Noteably, the ShlA/FhaA/HecA family adhesin/hemolysin (WP_004723375.1) was identified in 3/4 rainbow trout isolates, but only in a single Atlantic salmon isolate. Furthermore, the partial filamentous hemagglutinin (WP_004722541.1) was identified only in the rainbow trout isolates RD6 and RD124 (in two replicates). Several OMPs with roles in iron acquisition were also identified, including FiuA, FhuA, ShuA, HasR and other TonB-dependent receptors, although these were not present in all isolates and in all replicates. Two OmpC homologues, OmpC.1 and OmpC.2, were identified by both bioinformatic and proteomic approaches. The genes encoding these proteins were identified in all four genomes but OmpC.1 was detected only in three isolates and OmpC.2 in six isolates. BLAST alignment of these proteins demonstrated 54% sequence identity to each other.

Sixteen OMPs were identified solely in rainbow trout isolates. These included a partial filamentous hemagglutinin, LpoA/LppC, SecD, YajG, OmpW, MltC, PepM37, lipoprotein 1, Blc, a Type 3 secretion system (T3SS) protein, LptD, FhuA, and four hypothetical proteins (hypothetical proteins 15, 18, 19 and 20). However, most of these proteins were identified in only a single replicate of one isolate with the exception of the TTSS protein (RD6; two replicates), a partial filamentous hemagglutinin (RD6 and RD124; two replicates) and hypothetical protein 15 (RD6 and RD28; one and two replicates, respectively). Five OMPs were unique to isolates recovered from Atlantic salmon. These included a TpsB-family protein, FadL, RlpA, RupA and Phospholipase A1 although again these were typically present in only one replicate and one isolate with the exceptions of FadL (RD354 and RD420; one and two replicates, respectively) and the TpsB family protein (RD382 and RD420; one and two replicates, respectively). The proteins RlpA and phospholipase A1 were both identified in single replicates of isolate RD420 and RupA was identified in a single replicate of isolate RD366.

#### 3.3.2 Gel-based analysis

The major protein bands representing OmpA, OmpC and OmpF were excised and analysed to confirm their identity with respect to their designated OMP-types [20]. The identity of OmpA, OmpC and OmpF was confirmed in each isolate, endorsing the reliability of the OMP-based typing scheme. Excision and analysis of specific protein bands that were not visible in the OMP profile of isolate RD366, but were present in one or more of the other seven isolates, identified four previously unidentified OMPs (Fig. S2 and Table S3). Thus, YiaD/Omp16 (protein 20) was identified in isolates RD28, RD382 and RD420, RlpA (protein 38) in isolate RD382, Surface Ag (protein 41) in isolate RD382 and MalA (protein 42) in isolates RD28 and RD382. Of these proteins, YiaD/Omp16 (all isolates) and RlpA (isolate RD420 only) were also identified using gel-free proteomics (Table S2). It should be noted that these four proteins were not identified in isolate RD366 using either the L-S or I-B approaches (Table 3), although YiaD/Omp16 and RlpA were identified by the gel-free approach.

#### 3.3.3 Comparison between bioinformatic prediction and proteomic identification

A total of 84 OMPs were identified among the eight *Y. ruckeri* isolates using a combination of gel-free and gel-based proteomic approaches (Table S4). However, only 60 (71.4%) of these had been predicted to be OMPs using bioinformatic prediction. Of the 24 (28.5%) OMPs that were identified by proteomics but not predicted, four (HemR, TcyP, MalA and DuaA) were involved in transport, one (hypothetical protein 16) had enzymatic activity, one (filamentous hemagglutinin) was involved in adhesion, eight had other functions (OsmY, HslJ, YfhG/QseG, TpsB family, YegD/Hsp70, GlpC, Lipoprotein 3 and Surface Ag), and 10 (YeeJ, YqjD, C-terminal protease, hypothetical proteins 15, 18, 19 and 20, RupA, Membrane protein and NlpD) were of unknown function. Conversely, 81 confidently predicted OMPs were not experimentally identified. These included 10 transmembrane β-barrel proteins, 27 outer membrane lipoproteins and 44 proteins predicted as both. These OMPs belonged to various functional categories including outer membrane biogenesis and integrity (two proteins), transport and receptor (14 proteins), adherence (13 proteins), enzymatic activity (nine proteins) and motility (five proteins). However, they also included 38 confidently predicted OMPs with other (15 proteins), hypothetical (11 proteins) or unknown (12 proteins) functions.

## 4 Discussion

In the present study, a combination of bioinformatic and complementary proteomic approaches were applied to analyse the OM proteomes of eight representative isolates of *Y. ruckeri* recovered from Atlantic salmon and rainbow trout. A bioinformatic workflow was first employed to identify outer membrane-localised proteins in four strains of *Y. ruckeri* from publicly available genomes (Table 1). Using this approach, 141 OMPs (representing 2.69 to 3.33% of the respective proteomes) were confidently predicted in the four strains (Table S1). These figures are lower than those obtained for the *P. multocida* avian outer membrane proteome (4.8%) [37] and for the outer membrane proteome of the intracellular pathogen *Ehrlichia ruminantium* (5.4%) [38], but are similar to figures obtained for the outer membrane proteomes of several members of the *Chlamydiae*, namely *Parachlamydia acantamoebae* (2.5%), *Simkania negevensis* (3.8%), and *Waddlia chonrophila* (2.8%) [39]. However, the genomes of *Y. ruckeri* analysed in the present study encode substantially more proteins (3079 to 3352) than do those of *P. multocida* (2009)*, E. ruminantium* (950) or any of the *Chlamydiae* genomes (1828 to 2531). Putative functions could be assigned to 121 (85.8%) of the 141 confidently predicted OMPs based on the published literature (Table S1). Forty eight OMPs (34%) were identified in all four genomes, although this increased to 77 (54.6%) when OMPs identified in at least three out of four genomes were considered. These 77 proteins were considered the ‘core’ outer membrane proteome.

Upon assigning functional categories to the predicted OMPs (Table S5), 15 (10.6%) were determined to have roles in outer membrane biogenesis and integrity. These included the proteins LptD and LptE which have roles in LPS assembly, Brauns lipoprotein (Lpp) which functions in anchoring the outer membrane to the peptidoglycan layer, and various members of the β-barrel assembly machinery (BAM) complex (BamA-E). Over one fifth (20.6%) of the predicted OMPs had roles in transport or as receptors. These included the porins OmpC, OmpE, OmpF, LamB and proteins involved in iron transport including FiuA, FhuA, ShuA, HasR and other TonB-dependent receptors. Sixteen (11.3%) of the predicted OMPs were involved in adherence. Of these, 14 were predicted to have roles in fimbriae or pili assembly, although only FimD and PilF were identified in all four genomes. Fimbrial adhesins play important roles in adherence and colonisation of other fish pathogenic bacteria, including *E. tarda* and *Aeromonas hydrophila* [73,74], although their role in the pathogenesis of *Y. ruckeri* has yet to be elucidated. Nineteen proteins (13.5%) were involved in enzymatic activities of which eight were identified in all four genomes. Ten proteins (7.1%) were involved in motility and these were identified in all four genomes examined. Twenty-one proteins were predicted to have other functions and 17 proteins had unknown functions whereas there were 14 hypothetical proteins.

Gel-free and gel-based proteomic approaches were subsequently used to identify those proteins expressed in the outer membrane of *Y. ruckeri* when grown under standard laboratory conditions. While gel-free proteomics is useful for the analysis of complex samples, one-dimensional (1-D) SDS-PAGE has advantages because hydrophobic membrane proteins are solubilised and unfolded under the denaturing conditions used [75] and multiple samples can be run and compared on the same gel. Although 1-D SDS-PAGE has lower resolution of protein separation than that of 2-D PAGE, the latter has limitations in its ability to separate hydrophobic proteins [34,63]. Special consideration was given to the method of protein extraction. Sarkosyl selectively solubilises the inner membrane and produces an insoluble fraction representing the outer membrane-peptidoglycan complex [76]. Hobb et al. [77] examined nine different methods to extract outer membrane-fractions of *Campylobacter jejuni* and concluded that Sarkosyl extraction provided the purest outer membrane extracts and was the most reproducible method. This method is commonly used to prepare outer membrane fractions of Gram-negative bacteria and “clean” outer membrane-enriched fractions are readily obtained [63,[78], [79], [80], [81], [82]]. For these reasons, outer membranes were extracted with Sarkosyl and 1-D SDS-PAGE was used for the gel-based approach.

As a first step, gel-free and gel-based proteomic analyses were conducted on a single representative isolate, RD366, to allow these two approaches to be compared and contrasted as methods for providing optimum characterisation of the *Y. ruckeri* OM proteome. Thirty-eight OMPs were identified in isolate RD366 by gel-free analysis, of which 30 (78.9%) were predicted (Table 3). While several studies have utilised gel-free proteomics to identify bacterial proteins, the identification of OMPs with this technique is less common. Wurpel et al. [49] identified 47 OMPs from OMV fractions of *E. coli* whereas Choi et al. [48] identified 64 OMPs in OMV fractions of *Pseudomonas aeruginosa* and Stekhoven et al. [41] identified 74 OMPs from whole cell fractions of *Bartonella henselae*. A recent global gel-free proteomic profiling study identified 23 OMPs across four strains of *Y. ruckeri* [64]. These included the major OMPs, OmpA, OmpC and OmpF, the hydrophobic compound transporter OmpW, outer membrane assembly factors (BamA-E), outer membrane lipoproteins (Blc, pcp, RcsF, LolB, LolD, Omp16, RcsF and YfeY), lipoproteins (NlpD, NlpE and NlpI) and lipopolysaccharide biosynthesis proteins (LptA, LptD and LptE). Of these 23 OMPs, 15 were also identified in isolate RD366 in the present study (Table 3). The lipoproteins Blc, RcsF, LolB, LolD, RcsF, YfeY and NlpD and lipopolysaccharide biosynthesis protein LptA were not identified by either proteomic method. A meaningful comparison of the two studies is challenging because Kumar et al. (2016) utilised whole-cell proteomic analyses whereas our study was focused on purified outer membrane extracts. It is possible that outer membrane isolation by Sarkosyl extraction may result in the sheering of loosely associated outer membrane lipoproteins, making their identification through this approach more difficult. Although we failed to identify eight proteins that were described by Kumar et al. (2016), we nevertheless identified numerous additional OMPs that these authors did not identify and these findings highlighted the advantages of our approach.

Forty-seven OMPs were identified in isolate RD366 using the L-S gel-based approach (Fig. 4A; Table 3). The identification of 30 proteins in all three replicates and 38 proteins in at least two replicates highlighted the reproducibility of the method (Fig. 4B). Excision and analysis of only visible protein bands of isolate RD366 in three replicates using the I-B gel-based approach allowed identification of 41 OMPs (Fig. 4C). In this case, 29 proteins were common to all three replicates and 34 identified in at least two replicates (Fig. 4D). Each of these gel-based approaches confers different advantages; the L-S approach offering greater coverage and the I-B method allowing protein bands on the gel to be identified. Combining these two gel-based methodologies resulted in the identification of 53 unique OMPs (Fig. 4E), of which 45 (84.9%) were confidently predicted by bioinformatics (Table 3). Of previous studies that have utilised gel-based proteomics to identify OMPs, Watson et al. [55] identified 19 OMPs in *Lawsonia intracellularis*, Wang et al. [50] identified 19 OMPs in *E. tarda* and Ayalew et al. [52] identified 55 OMPs in *M. haemolytica*. Using 2-D gel-based proteomics, Coquet et al. [65] identified 55 OMPs in *Y. ruckeri* in a comparison of bacteria grown in planktonic culture and as a biofilm.

The application of both gel-free and gel-based proteomic approaches allowed the identification of 65 OMPs in isolate RD366 (Table 3). Whereas 26 proteins were identified using both methods, 12 proteins were identified exclusively using the gel-free approach and 27 proteins were identified only using the combined (L-S and I-B) gel-based approaches (Fig. 5). Although only 10 out of 38 proteins were identified in three biological replicates of the gel-free samples of isolate RD366, which was less than might be expected, the overlap increased to 20 proteins for two or more replicates. The OMP profiles of the replicates in SDS-PAGE gels were highly consistent (Fig. 4) and the gel-based analyses demonstrated very high uniformity of protein composition of the same samples (30/47 proteins were present in 3 replicates for the L-S approach and 29/41 proteins were present in 3 replicates for the I-B approach). We have also demonstrated higher levels of protein overlap for gel-free analysis of biological replicates of other bacterial species (*M. haemolytica* and *P. multocida*) and believe that the lower overlap observed for *Y. ruckeri* is a species-specific phenomenon. Clearly, the use of these complementary approaches resulted in improved overall coverage of the outer membrane proteome and was consistent with previous work in other bacterial species [44,[61], [62], [63],^83^,^84^].

While a combination approach increased the total number of OMPs identified in *Y. ruckeri*, the gel-based study identified more unique OMPs than the gel-free approach and, importantly, some of these OMPs have putative roles in virulence including proteins that have not previously been considered or identified as virulence factors in Y*. ruckeri*. These include proteins such as CsgG, TamA/YtfN, LptD, OmpW, Phos. A1, OmpX, PilV and GlpC, as well as the iron transport protein ShuA. CsgG is an outer membrane lipoprotein required for the secretion and stabilisation of CsgA and CsgB which form curli amyloid fibres on the extracellular surface [85]. These fibres have been implicated in a number of processes including biofilm formation, attachment and invasion of host cells, interaction with host proteins and activation of the immune system [85,86]. TamA/YtfN is involved in autotransporter assembly and ultimately adhesion, with deletion of *tamA* being shown to inhibit the virulence of several pathogens [87,88]. Autotransporters have recently been shown to be important in the pathogenesis of *Y. ruckeri* [89]. Transport of mature LPS to the outer membrane requires the LPS transport (Lpt) machinery [90]. The integral β-barrel protein LptD and lipoprotein LptE form a complex in the outer membrane and are required for the final stage of LPS assembly at the cell surface. OmpW belongs to the same family of proteins as OmpA, OmpX and other eight stranded β-barrel proteins [91] and is involved in the protection of bacteria against various forms of environmental stress, including osmosis [92], oxidation [93], temperature and the unavailability of nutrients and oxygen [94]. OmpW is also involved in bacterial resistance to antibiotics, including ampicillin, tetracycline and ceftriaxone [95], and increased bacterial survival during phagocytosis [96]. Phospholipase A1 has been implicated in the virulence of *E. coli*, *Campylobacter* and *Helicobacter* strains by allowing the release of colicins or virulence factors [29]. OmpX is structurally similar to OmpA in terms of its β-sheet topology and belongs to a family of highly conserved proteins that appear important for virulence by neutralising host defence mechanisms [29]. The prototype OmpX protein, Ail from *Y. enterocolitica*, promotes adhesion to, and entry into, eukaryotic cells [97]. PilV represents a type IV pilus-associated protein that is important for adherence in *Neisseria gonorrhoeae* [98] and *Yersinia* sp. [99]*.* GlpC, encoding the anaerobic glycerol-3-phosphate dehydrogenase subunit C, has been implicated in tolerance of organic solvents in *E. coli* [100]. Several OMPs with roles in iron acquisition were identified including ShuA, HemR and other TonB-dependent receptors. Iron is a component in the catalysis of key enzymatic reactions and is required for growth and virulence [101]. Previously, Davies [102] observed four OMPs (of 66, 68, 69.5 and 72 kDa) that were upregulated in a low-iron environment, while Romalde et al. [103] observed the upregulation of three proteins of approximate molecular masses of 69, 73 and 77 kDa. Our proteomic analysis (Table 3) demonstrated that ShuA (gel-based only) and HemR (gel-free only) have molecular masses of 73.4 and 73.0 kDa, respectively, and it is likely that these represent two of the proteins previously observed. However, further proteomic analysis under iron-depleted growth conditions is likely to be more revealing as to the identity and regulation of specific iron-uptake proteins. Similarly, Lipoprotein 3 and hypothetical proteins 12 and 20 were identified in all three replicates by gel-based approaches but were absent from the gel-free analysis. While no definitive function can be assigned to these proteins, they may have roles in virulence or host specificity. To date, none of the above-named proteins have been considered to play roles in the pathogenesis of *Y. ruckeri*. Therefore, the identification of these OMPs in *Y. ruckeri*, with putative roles in virulence in other bacterial species, could form the basis of future studies to elucidate their roles in the pathogenesis of ERM and yersiniosis.

The gel-based approach identified a larger number of proteins than the gel-free approach (53 *versus* 38), including a number of key proteins potentially involved in virulence. The major advantage of gel-based proteomic approaches over gel-free techniques is the additional fractionation step conducted through SDS-PAGE. This results in less complex samples which are easier to digest and analyse by MS/MS. However, there are significant financial and time costs associated with this technique, meaning that it is less well-suited to large scale studies involving multiple strains. In addition, it has also been shown that the gel-based approach may under-represent small lipoproteins and low abundance proteins which can be detected by the gel-free method [104]. For these reasons, and recognising the limitations, a gel-free proteomic approach was employed to assess and compare the outer membrane proteomes of a wider range of strains representing different biotypes, serotypes and OMP-types of *Y. ruckeri*. Sixty-four OMPs were identified in eight representative isolates of *Y. ruckeri* using the gel-free approach and 48 (75.0%) of these proteins were confidently predicted by bioinformatics (Table S2). This number was marginally less than the total number of proteins (65) identified in isolate RD366 using both approaches. The 16 proteins that were not predicted were nevertheless confirmed to be OMPs by subsequent BLAST analysis and literature searching. These proteins were most likely not identified by bioinformatic prediction either due to their absence in the genomes used for prediction, the high stringency of the prediction pipeline, or manual error. The number of proteins identified in the eight *Y. ruckeri* strains ranged from 33 (isolate RD382) to 46 (isolate RD6) (Table S2). Seven OMPs (BamB, Lpp [Brauns], Pal, OmpA, OmpF, BamA/YaeT and OsmY) were identified in all three replicates of all eight isolates (85% of which were core proteins [Table S1]). The flagellar proteins, flagellin (FlaA) and FlgE, were identified in almost all replicates of all isolates, with the exception of isolate RD6. This strain is a non-motile biotype 2 isolate and the absence of flagella, or flagella apparatus proteins, was not surprising. Welch et al. [105] demonstrated that the *fliP, fliQ, fliR, flhB, flhA* and *flhE* genes encoding flagellar biosynthesis proteins are present in biotype 2 isolates but carry various mutations resulting in gene truncation. Therefore, the proteins encoded by these genes are likely to be predicted within the biotype 2 genome but affected flagella proteins are likely to be absent or non-functional. The additional four proteins identified by a limited gel-based analysis of the eight isolates complemented the more extensive gel-free analysis. However, as we have shown above for isolate RD366 (Table 3), additional proteins would likely be identified in regions of the gel containing no visible protein bands.

A major objective of the present study was to identify OMPs associated specifically with Atlantic salmon or rainbow trout isolates. The identification of such proteins might provide clues about potential mechanisms of host-specificity within this bacterial species. The host-specificity of *Y. ruckeri* has recently been highlighted and reinforced by Gulla et al. (2018). Based on the gel-free analysis, 16 OMPs were identified solely in isolates recovered from rainbow trout (RD6, 28, 64 and 124). These included filamentous hemagglutinin, LpoA/LppC, SecD, YajG, OmpW, MltC, PepM37, lipoprotein 1, Blc, a TTSS protein, LptD, FhuA, and four hypothetical proteins (15, 17, 18 and 19). Conversely, five OMPs were unique to isolates recovered from Atlantic salmon (RD354, 366, 382 and 420); these included a TpsB-family protein, FadL, RlpA, RupA and Phospholipase A1. Some of these proteins represent putative virulence factors in rainbow trout or Atlantic salmon. Of the rainbow trout-associated proteins, filamentous hemagglutinins are involved in general adhesion to host cells [106,107]; PepM37 represents a family of metallopeptidases involved in the enzymatic degradation of peptides [108]; FhuA is the receptor for the siderophore ferrichrome and is involved in the uptake of iron [109,110]; the presence of a TTSS protein is in agreement with the identification of a complete TTSS locus in *Y. ruckeri* [70] which likely plays a role in invasion and intracellular survival [70,111]; proteins of unknown or hypothetical function (YajG, hypothetical proteins 15, 17, 18 and 19) could be involved in virulence and warrant further study. Of the Atlantic salmon-specific proteins, TpsB-family proteins have potential roles in hemolysin and autotransporter activities [[112], [113], [114]]; RupA is involved in uptake of the siderophore ruckerbactin [115]; and PhosA1 (Phospholipase A_1_), an enzyme that hydrolyzes phospholipids and produces 2-acyl-lysophospholipids and fatty acids, plays an important role as a virulence factor in *Y. enterocolitica* [116,117]. Since none of these proteins were recovered from all of the isolates representing each host species or from all of the replicates representing each isolate, the data do not demonstrate a strong correlation between any specific protein and host-specificity. However, as discussed above, the gel-free proteomic approach was not as effective as the gel-based approach and use of the latter methodology might be more productive in identifying such proteins. In addition, isolates recovered from different host species may encode the same proteins but differences in gene regulation, amino acid sequence or post-translational modifications are also likely be involved in host-specificity [[118], [119], [120], [121], [122], [123]]. Therefore, further comparative studies designed to assess nucleotide and amino acid sequence variation, gene regulation and post-translational modification of proteins might be required to address the molecular basis of host-specificity in *Y. ruckeri*.

In conclusion, we have identified 84 OMPs (Table S4) expressed in *Y. ruckeri* under standard laboratory conditions. Some of these proteins have potential roles in the pathogenesis of *Y. ruckeri* and could form the basis of further studies aimed at the development of improved vaccines. In particular, cross-protective antigens could be identified by reverse vaccinology [[124], [125], [126]] and confirmed using immunoproteomic approaches [127,128]. Such studies could also be linked with the analysis of OMP expression under *in vivo* or *in vivo*-like growth conditions: *in vivo* proteomics have been utilised previously to examine the outer membrane proteome of *Aeromonas salmonicida* grown in dialysis tubing implanted into Atlantic salmon [129], the outer membrane proteome of *P. multocida* recovered from the bloodstream of infected chickens [34], and OMPs upregulated in *A. salmonicida* grown in an *in vivo* growth chamber model [130]. Thus, further analysis of *Y. ruckeri* isolates grown *in vivo*, or under growth conditions replicating those *in vivo* [131,132], is likely to identify additional proteins involved in virulence and host-adaptation and such findings will have important implications for improving future vaccination and control strategies.

The following are the supplementary data related to this article.

Supplementary material Image 1

## Funding

This work, including the efforts of Michael J. Ormsby, was funded by an Industrial CASE PhD studentship award from the Biotechnology and Biological Sciences Research Council (BBSRC; BB/I01554X/1). Additional financial support was provided by the Centre for Environment, Fisheries and Aquaculture Science (Cefas), Ridgeway Biologicals Ltd., Marine Harvest Scotland, and Dawnfresh Seafoods. The BBSRC had no role in study design, data collection and interpretation, or the decision to submit the work for publication.
