# Supplementary material for: Comparative bioinformatic and proteomic approaches to evaluate the outer membrane proteome of the fish pathogen Yersinia ruckeri
Source: J Proteomics. 2019 May 15;199:135–47. doi: 10.1016/j.jprot.2019.02.014 (PMC6447952; doi:10.1016/j.jprot.2019.02.014)
Supplement: Supplementary file 1 — Supplementary material [file mmc1.pdf]

SUPPLEMENTARY INFORMATION

Comparative bioinformatic and proteomic approaches to evaluate the outer membrane proteome of the fish pathogen *Yersinia ruckeri*

Michael J. Ormsby[a], Edward Grahame[a], Richard Burchmore[a,b] and Robert L. Davies[a*].

[a]*Institute of Infection, Immunity and Inflammation, College of Medical, Veterinary and Life Sciences, Sir Graeme Davies Building, University of Glasgow, Glasgow G12 8TA, UK*

[b]*Polyomics, Institute of Infection, Immunity and Inflammation, College of Medical, Veterinary and Life Sciences, TCRC, University of Glasgow, Glasgow G12 1QH*

**Corresponding author email: Robert.Davies@glasgow.ac.uk*

CONTENTS

Fig. S-1 Schematic depicting the gel-free and gel-based techniques for outer membrane protein identification.

Fig. S-2 The excised bands from OMP profiles of representative isolates of *Y. ruckeri* separated by 1D SDS-PAGE.

Table S-1 List of confidently predicted OMPs from four publicly available genomes of *Y. ruckeri.*

Table S-2 Proteins identified by gel-free proteomic analysis of eight isolates of *Y. ruckeri*.

Table S-3 Additional OMPs identified through gel-based proteomics of *Y. ruckeri.*

Table S-4 Proteins identified in eight isolates of *Y. ruckeri* through a combination of gel-free and gel-based proteomic techniques.

Table S-5 Functional categories of 141 predicted OMPs in four *Y. ruckeri* genomes.

Fig. S-1 Schematic depicting the gel-free and gel-based techniques for outer membrane protein identification. The outer membrane was extracted and enriched by Sarkosyl extraction (A). Each independent outer membrane extract was divided into three portions and analysed by (B) gel-free and gel-based (individual band [I-B; C.1] and lane-section [L-S; C.2]) proteomic techniques. In the I-B format individual protein bands were excised, while in the L-S format the entire lane was excised. Both gel-free and gel-based samples were subjected to trypsin digestion (D), analysed by LC-MS/MS (E) and the proteins subsequently identified using MASCOT and literature searching (F).

Fig. S-2 The excised bands from OMP profiles of representative isolates of *Y. ruckeri* separated by 1D SDS-PAGE. Isolates RD6, RD28, RD64, RD124 (recovered from Rainbow trout); and isolates RD354, RD366, RD382 and RD420 (recovered from Atlantic salmon) are in the order indicated. Three replicates are represented by 1, 2 and 3. Lane 7 of each panel represents a MW standard (GE Healthcare, UK). Identified proteins are labelled numerically and correspond to Tables S-3 and S-4. Numbers labelled in red indicate proteins that were uniquely identified using gel based methods.

Table S-1 List of confidently predicted OMPs from four publicly available genomes of *Y. ruckeri*.

	Protein	Function	MW (Da)	Genome (GenBank Accession number)				Location			Core proteins[d]
				ATCC29473	CSF007-82	37551	YRB	1[a]	2[b]	3[c]	
**1. OM biogenesis and integrity**											
1	BamA/YaeT/Omp85	BAM complex	88100	KGA50688.1	CEK28575.1	KFE37550.1	AJI95279.1	+	+	+	+
2	BamB	BAM complex	42300	KGA51489.1	CEK28417.1	KFE40157.1	AJI96267.1	-	+	+	+
3	BamC/NlpB	BAM complex	38300	KGA51611.1	CEK28370.1	KFE40200.1	AJI95961.1	+	+	+	+
4	BamD/YfiO	BAM complex	27400	KGA49534.1	CEK26610.1	KFE37895.1	AJI93676.1	-	-	+	+
5	BamE/SmpA	BAM complex	12500	KGA51354.1	CEK28487.1	KFE40087.1	AJI95242.1	-	-	+	+
6	LolB/HemM	Chaperone and lipoprotein transport	24200	KGA49330.1	CEK27340.1	KFE39446.1	-	-	-	+	+
7	Lpp (Brauns)	Anchors peptidoglycan to OM	8400	KGA51422.1	CEK27669.1	KFE39275.1	AJI94418.1	-	-	+	+
8	LptD/Imp	LPS assembly	89700	KGA50232.1	CEK26380.1	KFE40530.1	AJI93953.1	+	+	+	+
9	LptE/RlpB	LPS assembly	21000	KGA49081.1	CEK26865.1	KFE38612.1	AJI94665.1	-	-	+	+
10	OmpA	OM integrity, porin, adherence	37800	KGA49573.1	CEK27184.1	KFE40006.1	AJI95876.1	+	+	+	+
11	Pal	Peptidoglycan associated	18100	KGA49271.1	CEK26938.1	KFE37976.1	AJI95644.1	-	-	+	+
12	Slp family	Structural and OM stabilisation	20600	KGA50105.1	-	KFE39499.1	AJI94566.1	-	-	+	+
13	SlyB	Membrane integrity	15300	KGA50971.1	CEK27645.1	KFE39255.1	AJI95837.1	-	-	+	+
14	VacJ/MlaA	Phospholipid homeostasis	28800	KGA50830.1	CEK28278.1	KFE37380.1	AJI93751.1	-	-	+	+
15	YbhG	Membrane anchor protein	35700		CEK26984.1	KFE38061.1	-	+	+	+	
**2. Transport and receptor**											
16	Blc	Lipid storage/transport	20600	-	CEK26156.1	-	-	-	-	+	
17	BtuB	Vitamin B12 transport	69900	KGA46996.1	CEK29186.1	KFE37752.1	AJI94185.1	+	+	+	+
18	CsuB	Fimbrial chaperone (Type I pilus)	18600	KGA43942.1	-	-	-	+	-	-	
19	CsuC	Fimbrial chaperone (Type I pilus)	28700	KGA44131.1	-	KFE39562.1	AJI95148.1	-	-	+	+

Table S-1 Continued

	Protein	Function	MW (Da)	Genome (GenBank Accession number)				Location			Core proteins[d]
				ATCC29473	CSF007-82	37551	YRB	1[a]	2[b]	3[c]	
20	CsuD	Fimbrial usher (Type I pilus)	86400	KGA44132.1	-	-	AJI96073.1	+	+	-	
21	CsuE-like	Surface-fimbriae tip adhesin	34500	-	CEK25859.1	KFE39564.1	AJI93735.1	+	-	+	+
22	FadL/OmpP1	Fatty acid/Hydrophobic compound trans.	46100	KGA51106.1	CEK28277.1	KFE37381.1	AJI96387.1	+	+	+	+
23	FhuA	Ferric hydroxymate receptor	81700	KGA50060.1	CEK28777.1	KFE37693.1	AJI95410.1	+	+	+	+
24	FiuA	Iron transport	78400	KGA49280.1	-	KFE38314.1	AJI93637.1	+	+	+	+
25	HasR	Iron transport	93100	KGA47003.1	CEK29201.1	KFE37766.1	AJI96066.1	+	+	-	+
26	LamB	Maltoporin	47600	KGA43775.1	CEK28986.1	KFE40355.1	AJI96368.1	+	+	+	+
27	MalE	Maltose export	43800	KGA43809.1	-	-	-	-	-	+	
28	OmpC.1	Porin (Small molecules)	40200	KGA49862.1	CEK28175.1	KFE39990.1	AJI96611.1	+	+	+	+
29	OmpC.2	Porin (Small molecules)	40000	KGA51002.1	CEK27297.1	KFE39406.1	AJI96049.1	+	+	+	+
30	OmpE	Inorganic phosphate uptake	39900	KGA49176.1	CEK27010.1	KFE38036.1	AJI94786.1	+	+	+	+
31	OmpF	Porin (Small molecules)	41400	KGA49458.1	CEK27167.1	KFE38186.1	AJI94673.1	+	+	+	+
32	OmpL/KdgM	Porin (oligogalacturonides)	26700	KGA49549.1	-	-	AJI96240.1	+	+	+	
33	OmpW	Small hydrophobic compound transport	23300	-	CEK27485.1	KFE39106.1	AJI94704.1	+	+	+	+
34	OmpX	Virulence related	18400	KGA49040.1	CEK27020.1	KFE38026.1	AJI96167.1	+	+	+	+
35	OprC	Copper Receptor	81400	KGA44083.1	-	-	AJI93749.1	+	+	+	
36	OprD	Chitoporin	48400	KGA49446.1	-	-	AJI94954.1	+	+	+	
37	PhnE	Phosphonate ABC transporter	23300	-	CEK27752.1	KFE39356.1	-	-	+	+	
38	ShuA	Heme receptor	74400	KGA51659.1	CEK27685.1	KFE39290.1	AJI93789.1	+	+	+	+
39	Skp	Chaperone	18300	KGA50681.1	-	-	-	-	-	+	
40	TamA/YtfN	Autotransporter assembly	64800	KGA49196.1	CEK26208.1	KFE39791.1	AJI93607.1	+	+	+	+
41	TctC	Tricarboxylic transport	35900	-	CEK26742.1	KFE38402.1	AJI95284.1	-	-	+	+

Table S-1 Continued

	Protein	Function	MW (Da)	Genome (GenBank Accession number)				Location			Core proteins[d]
				ATCC29473	CSF007-82	37551	YRB	1[a]	2[b]	3[c]	
42	TolC	OM Channel (Efflux system)	52800	KGA50323.1	CEK28812.1	KFE37729.1	AJI94078.1	+	+	+	+
43	TonB dependent receptor	Iron transport	76600	KGA49840.1	CEK26970.1	KFE38074.1	AJI94667.1	+	+	+	+
44	Tsx	Colicin/phage receptor	29100	KGA49816.1	CEK26700.1	KFE38363.1	AJI93985.1	+	+	+	+
**3. Adherence**											
45	AidA	Adhesin	91700	KGA49140.1	CEK28856.1	KFE40228.1	AJI94485.1	-	-	+	+
46	Autotransporter	Adhesin	78200	KGA50029.1	CEK26284.1	KFE37415.1	AJI96502.1	+	+	+	+
47	FimA	Fimbrial protein	17900	KGA49679.1	-	-	-	-	-	+	
48	FimD	FimA export and assembly	96800	KGA43941.1	CEK28395.1	KFE38567.1	AJI93657.1	+	+	+	+
49	PefC	Fimbrial usher	86700	KGA49043.1	-	KFE39434.1		+	+	+	
50	PilF	Minor pilin	27300	KGA51554.1	CEK28422.1	KFE40152.1	AJI96335.1	+	-	+	+
51	PilP	Type IV pilus bio.	20600	KGA44935.1	-	-	-	+	-	+	
52	PilQ	Type IV pilus biogenesis	82500	KGA43940.1	CEK26012.1	-	AJI95021.1	+	+	-	+
53	PilV	Minor pilin	42700	KGA44898.1	-	-	-	+	-	-	
54	PqiB/Mam7	Adherence	60600	-	CEK27178.1	KFE40000.1	-	+	-	-	
55	ShlA/HecA/FhaA family	Heme utilization and adhesin	418400	KGA49502.1	CEK28851.1	KFE40224.1	-	+	+	+	+
56	SteB	Fimbral protein	98200	KGA49062.1	CEK28007.1	KFE38435.1	-	+	-	+	+
57	SteJ	Fimbrial usher	79700	KGA49225.1	CEK26187.1	-	-	+	+	+	
58	TraL	F Pilin formation	15800	KGA44942.1	-	-	-	-	-	+	
59	Fimbral family protein	Fimbrial bioproteinsis usher	31400	KGA49295.1	-	-	-	+	+	-	
60	SfmD	Fimbrial usher	92300	KGA50881.1	-	KFE40177.1	AJI94903.1	+	+	+	+
**4. Enzymatic activity**											
61	BcsC	Cellulose biosynthesis	128800	KGA44060.1	CEK25913.1	KFE39041.1	AJI96493.1	-	-	+	+
62	Hemolysin	Hemolysin	157800	KGA49858.1	-	KFE39468.1	AJI93845.1	+	+	+	+
63	LpoA	Peptidoglycan synthesis	70300	KGA43851.1	CEK28880.1	KFE40251.1	AJI94026.1	+	-	+	+

Table S-1 Continued

	Protein	Function	MW (Da)	Genome (GenBank Accession number)				Location			Core proteins[d]
				ATCC29473	CSF007-82	37551	YRB	1[a]	2[b]	3[c]	
64	LpoB	Peptidoglycan synthesis	20600	KGA50017.1	CEK27239.1	KFE40058.1	-	-	-	+	+
65	MetQ	D-methionine binding	29300	KGA50649.1	CEK28552.1	KFE37527.1	AJI95826.1	-	-	+	+
66	MltA	Peptidoglycan maintenance	42900	KGA50559.1	CEK28603.1	KFE40370.1	AJI96420.1	+	-	+	+
67	MltB	Peptidoglycan maintenance	39200	KGA51419.1	CEK28291.1	KFE37367.1	AJI96166.1	+	-	+	+
68	MltC	Peptidoglycan maintenance	39900	KGA50598.1	CEK28718.1	KFE40381.1	AJI93812.1	-	+	+	+
69	MltD	Peptidoglycan maintenance	52800	-	CEK26616.1	-	AJI95285.1	+	-	-	
70	MltE	Peptidoglycan maintenance	23800	-	CEK27726.1	-	-	-	-	+	
71	NlpC	Copper homeostasis	25300	KGA51327.1	CEK28556.1	KFE37531.1	AJI96307.1	-	-	+	+
72	PepM37	Enzymatic activity	50500	-	CEK29247.1	KFE37810.1	-	+	+	+	
73	Phoslipase A1	Bacteriocin secretion	33700	KGA44966.1	CEK29130.1	KFE38782.1	AJI95776.1	+	+	+	+
74	PrtF	Protease secretion	50800	KGA49670.1	-	KFE39387.1	-	+	+	+	
75	ShlB	Hemolysin activator	61900	KGA43827.1	-	-	AJI94208.1	+	+	+	
76	Spr	Enzymatic activity	21700	KGA51130.1	-	-	-	+	-	+	
77	YfgC	Zinc Metalloprotease	53900	-	CEK28375.1	KFE40195.1	-	+	-	+	
78	YhlA	Hemolysin	169600	-	CEK28999.1	KFE40367.1	-	+	-	+	
79	Endopeptidase	Enzymatic activity	16600	-	-	KFE37434.1	AJI94199.1	+	-	+	
**5. Motility**											
80	FlaA	Flagellin	44000	KGA51310.1	CEK27960.1	KFE39630.1	AJI94147.1	+	-	-	+
81	FlgD	Flagellar hook assembly	24200	KGA51491.1	CEK27994.1	KFE39661.1	-	+	-	-	+
82	FlgE	Links flagella to driver	44200	-	CEK27993.1	KFE39660.1	-	+	-	-	
83	FlgG	Distal rod protein	27900	KGA51309.1	CEK27991.1	KFE39658.1	-	+	-	-	+
84	FlgH	Flagella L-ring protein	25300	KGA51261.1	CEK27989.1	KFE39657.1	AJI94823.1	+	+	-	+

Table S-1 Continued

	Protein	Function	MW (Da)	Genome (GenBank Accession number) ATCC29473	CSF007-82	37551	YRB	Location 1[a]	2[b]	3[c]	Core proteins[d]
85	FlgI	Flagella P-ring protein	36700	KGA51583.1	-	-	AJI94007.1	+	-	+	
86	FlgK	Hook associated - filament junction	58000	KGA51150.1	CEK27986.1	KFE39654.1	AJI95156.1	+	-	+	+
87	FlgL	Hook associated - filament junction	35200	KGA50945.1	CEK27985.1	KFE39653.1	-	+	-	-	+
88	FliD	Flagellar filament cap	49100	-	CEK27963.1	KFE39631.1	AJI96018.1	+	-	-	+
89	FliF	Hook length control	51300	-	CEK27977.1	-	-	+	-	-	
**6. Unknown**											
90	Lipoprotein 2	Unknown	23300	KGA50955.1	-	-	AJI94414.1	-	+	+	
91	Lipoprotein 1	Unknown	20200	-	-	-	AJI93913.1	-	-	+	
92	OM Beta barrel	Unknown	22800	KGA50421.1	CEK27255.1	-	AJI96085.1	+	+	+	+
93	Smp	Unknown	25600	KGA50081.1	-	-	AJI94887.1	-	-	+	
94	YajG	Unknown	21000	KGA49401.1	-	-	AJI93736.1	-	-	+	
95	YbfN/ChiQ	Unknown	12000	KGA50145.1	-	-	AJI94103.1	-	-	+	
96	YceB	Unknown	20500	-	-	-	AJI94074.1	-	-	+	
97	YcfL	Unknown	14100	-	CEK27238.1	KFE40057.1	-	+	-	+	
98	YdgH	Unknown	34000	-	CEK27580.1	KFE39195.1	-	+	-	+	
99	YdiY-like	Salt induced OM protein	28000	KGA49863.1	CEK26603.1	KFE37902.1	AJI93661.1	+	-	+	+
100	YebT	Unknown	95100	-	-	KFE39349.1	-	+	+	-	
101	YfaZ	Unknown	18700	KGA51571.1	CEK28195.1	KFE38203.1	AJI93691.1	+	-	+	+
102	YfeY	Unknown	20900	KGA51263.1	-	-	AJI93981.1	-	-	+	
103	YgiB	Biofilm formation	23900	-	-	KFE37730.1	AJI93763.1	-	-	+	
104	YiaF	Unknown	25700	-	-	-	AJI94102.1	-	-	+	
105	YiiQ	Unknown	21800	-	-	KFE37795.1	-	+	+	+	
106	YjeI	Unknown	14200	KGA44079.1	-	-	-	-	+	+	
**7. Other**											
107	CsgG	Curli organelles and biofilm formation	24000	KGA51111.1	CEK27754.1	KFE39358.1	-	+	+	+	+

Table S-1 Continued

	Protein	Function	MW (Da)	Genome (GenBank Accession number)				Location			Core proteins[d]
				ATCC29473	CSF007-82	37551	YRB	1[a]	2[b]	3[c]	
108	GspD	General secretion path.	71400	KGA49459.1	CEK26578.1	KFE37926.1	AJI96597.1	+	+	+	+
109	HmsP	Biofilm formation	75500	-	CEK25914.1	KFE39040.1	-	-	-	+	
110	Invasin 1	Invasion	93100	KGA44022.1	CEK25926.1	-	-	+	+	+	
111	Invasin 2	Invasion	69400	-	-	KFE40369.1	-	+	+	+	
112	MipA	MltA interacting	28000	KGA49714.1	CEK27425.1	KFE39524.1	AJI95499.1	+	+	+	+
113	NlpI	Cell division	33800	KGA49038.1	CEK26241.1	KFE39758.1	-	-	-	+	+
114	OsmB	Osmotically inducible	6800	-	CEK27515.1	KFE39134.1	AJI96131.1	-	-	+	+
115	OutS	Secretion	14500	-	CEK26567.1	KFE37937.1	-	-	-	+	
116	RcpA	Pilus secretion	48300	-	CEK28783.1	KFE37699.1	-	-	+	+	
117	RcsF	Phosphorelay signaling path	14500	KGA50578.1	CEK28553.1	KFE37528.1	-	-	-	+	+
118	RlpA	Rod shape and daughter cell seperation	37600	KGA50174.1	CEK26858.1	KFE38505.1	AJI95197.1	+	-	+	+
119	TTSS protein	TTSS protein	45900	-	-	-	AJI96607.1	+	-	-	
120	TadD	Pilus assembly	28500	-	-	KFE37706.1	-	-	-	+	
121	TraN	F Plasmid conj. Transfer	35600	KGA44907.1	-	-	-	-	-	+	
122	YdgA	Serine protease	29300	-	-	-	AJI93679.1	-	-	+	
123	YiaD/Omp16	Multicopy suppressor of BamD	22200	-	CEK25848.1	KFE39573.1	-	+	+	+	
124	YnbE	YnbE-like	7400	KGA51004.1	-	-	-	-	-	+	
125	Yrap	Phospholipid biogenesis	20200	-	CEK28883.1	KFE40254.1	AJI96212.1	+	-	+	+
126	YscC	Type III secretion OM pore	63000	-	CEK26541.1	KFE37959.1	AJI94017.1	+	+	+	+
127	YscJ	Yop protein export	27500	-	CEK26536.1	KFE37964.1	AJI93682.1	+	-	+	+

Table S-1 Continued

	Protein	Function	MW (Da)	Genome (GenBank Accession number)				Location			Core proteins[d]
				ATCC29473	CSF007-82	37551	YRB	1[a]	2[b]	3[c]	
**8. Hypothetical**											
128	Hypothetical 12	Hypothetical	10800	-	-	-	AJI94018.1	-	-	+	
129	Hypothetical 10	Hypothetical	12200	-	CEK27624.1	KFE39235.1	-	+	-	+	
130	Hypothetical 9	Hypothetical	12200	-	CEK27596.1	KFE39209.1	-	+	-	+	
131	Hypothetical 14	Hypothetical	22900	-	-	KFE40074.1	AJI93929.1	+	+	+	
132	Hypothetical 7	Hypothetical	22900	KGA49308.1	-	KFE39813.1	AJI93964.1	+	+	+	+
133	Hypothetical 4	Hypothetical	24600	KGA51273.1	-	-	-	+	-	+	
134	Hypothetical 6	Hypothetical	26400	-	CEK28680.1	KFE40416.1	AJI94033.1	+	-	+	+
135	Hypothetical 8	Hypothetical	26500	-	CEK28711.1	KFE40387.1	-	+	-	+	
136	Hypothetical 1	Hypothetical	30000	-	CEK27557.1	KFE39173.1	AJI93918.1	+	+	+	+
137	Hypothetical 5	Hypothetical	33500	KGA49345.1	CEK26826.1	KFE38476.1	AJI93943.1	+	+	-	+
138	Hypothetical 13	Hypothetical	35300	KGA49328.1	CEK26283.1	KFE37414.1	-	+	+	+	+
139	Hypothetical 11	Hypothetical	50300	-	-	-	AJI93705.1	+	-	+	
140	Hypothetical 3	Hypothetical	75200	KGA43980.1	-	KFE39064.1	-	+	+	+	
141	Hypothetical 2	Hypothetical	80800	KGA44017.1	-	KFE39572.1	-	-	+	+	
			**Total**	**97**	**93**	**102**	**88**	**94**	**63**	**122**	**77**

[a]Proteins identified by subcellular localisation prediction programmes; [b]Proteins identified by β-barrel prediction programmes; [c]Proteins identified by lipoprotein prediction programmes; [d]Proteins predicted in at least three out of four genomes representing the ‘core’ outer membrane proteome.

Table S-2 Proteins identified by gel-free proteomic analysis of eight isolates of *Y. ruckeri*.

	NCBI RefSeq number	Protein	Function	MW (Da)	Rainbow trout				Atlantic salmon				Prediction
					RD6	RD28	RD64	RD124	RD354	RD366	RD382	RD420	
1	WP_004717998.1	BamB	BAM complex	42,328	+	+	+	+	+	+	+	+	+
2	WP_004700255.1	Lpp (Brauns)	Anchors peptidoglycan to OM	6,687	+	+	+	+	+	+	+	+	+
3	WP_004722151.1	Pal	Peptidoglycan associated	18,103	+	+	+	+	+	+	+	+	+
4	WP_004718926.1	OmpA	OM integrity, porin, adherence	37,756	+	+	+	+	+	+	+	+	+
5	WP_071704305.1	OmpF	Porin (Small molecules)	40,049	+	+	+	+	+	+	+	+	+
6	WP_004719660.1	BamA/YaeT	BAM complex	88,077	+	+	+	+	+	+	+	+	+
7	WP_004719968.1	OsmY	Osmoregulation	11,017	+	+	+	+	+	+	+	+	-
8	WP_038251462.1	Flagellin (FlaA)	Flagellin	43,615	-	+	+	+	+	+	+	+	+
9	WP_004720730.1	FlgE	Links flagella to driver	44,281	-	+	+	+	+	+	+	+	+
10	WP_004717911.1	VacJ/MlaA	Phospholipid homeostasis	28,735	+	+	+	+	+	+	+	+	+
11	WP_004719617.1	MetQ	D-methionine binding	29,316	+	+	+	+	+	+	+	+	+
12	WP_004723390.1	YeeJ	Unknown	54,668	+	+	+	+	+	+	+	+	-
13	WP_004717779.1	TcyP	Cysteine transporter	48,096	+	+	+	+	+	+	+	+	-
14	WP_004722544.1	ShlB/FhaC/HecB family	Hemolysin activator	62,461	+	+	+	+	+	+	+	+	+
15	WP_004722316.1	BamC	BAM complex	38,311	+	+	+	+	+	+	+	+	+
16	WP_004717609.1	Pcp/SlyB	Membrane integrity	15,334	+	+	+	+	+	+	+	+	+
17	WP_004717812.1	OmpC.1	Porin (Small molecules)	40,484	+	+	-	-	-	+	-	-	+
18	WP_004717101.1	OmpE	Inorganic phosphate uptake	39,949	+	+	+	+	+	+	+	+	+
19	WP_004723240.1	BcsC	Cellulose biosynthesis	126,490	+	+	+	+	+	+	+	+	+

Table S-2 Continued

	NCBI RefSeq number	Protein	Function	MW (Da)	Rainbow trout				Atlantic salmon				Prediction
					RD6	RD28	RD64	RD124	RD354	RD366	RD382	RD420	
20	WP_004719814.1	Slp Family	Structural and OM stabilisation	20,645	+	-	-	+	+	+	+	+	+
21	WP_004723105.1	BamD	BAM complex	27,392	+	+	+	+	+	+	-	-	+
22	WP_004719859.1	MipA	MltA interacting	28,040	+	+	+	+	-	+	+	+	+
23	WP_004722195.1	YqjD	Unknown	26,277	+	+	-	+	+	+	+	+	-
24	WP_004718264.1	YiaD/Omp16	Multicopy suppressor of BamD	22,248	+	+	+	+	+	+	+	+	+
25	WP_004719715.1	MltA	Peptidoglycan maintenance	42,866	+	+	+	+	+	+	+	+	+
26	WP_004723357.1	C-terminal protease	Unknown	75,933	+	+	+	+	-	+	-	+	-
27	WP_004720193.1	LptE/RlpB	LPS assembly	21,089	+	+	+	+	+	+	-	+	+
28	WP_004723375.1	ShlA/FhaA/HecA family	Adhesin	155,563	+	+	+	-	+	-	-	-	+
29	WP_004717528.1	HslJ	Heat-inducible protein	15,597	+	+	+	+	+	+	+	+	-
30	WP_004721031.1	TolC	OM Channel (Efflux system)	53,080	+	+	+	+	+	+	+	+	+
31	WP_004718068.1	YfhG/QseG	Quorum sensing/Virulence	34,809	+	+	+	+	+	+	+	+	-
32	WP_004720126.1	Hypothetical 5	Unknown	31,358	+	+	-	+	+	+	-	-	+
33	WP_004721707.1	CsgG	Curli organelles/biofilm formation	24,670	+	-	+	-	+	-	-	-	+
34	WP_004722541.1	Filamentous hemagglutinin, partial[a]	Filamentous haemaglutinnin	48,764	+	-	-	+	-	-	-	-	-
35	WP_004723020.1	TamA/YtfN	Autotransporter assembly	63,376	+	-	-	+	+	-	+	+	+
36	WP_004720881.1	ShuA	Heme receptor	73,397	+	-	-	+	-	-	+	-	+
37	WP_004722118.1	TTSS protein[a]	Type III secretion protein	61,640	+	-	-	-	-	-	-	-	+
38	WP_004717487.1	Hypothetical 15[a]	Unknown	34,982	+	+	-	-	-	-	-	-	-

Table S-2 Continued

	NCBI RefSeq number	Protein	Function	MW (Da)	Rainbow trout RD6	RD28	RD64	RD124	Atlantic salmon RD354	RD366	RD382	RD420	Prediction
39	WP_004717681.1	HemR	Iron transport	72,952	+	+	+	+	+	+	+	-	-
40	WP_004719473.1	BtuB	Vitamin B12 transport	68,999	+	+	+	-	-	+	+	-	+
41	WP_004718497.1	OprC	Chitoporin	75,008	+	+	-	+	+	+	+	+	+
42	WP_004720628.1	OmpC.2	Porin (Small molecules)	39,971	+	+	-	+	-	+	+	+	+
43	WP_004718917.1	PqiB/MAM7	Adherence	59,670	+	-	+	-	+	-	-	-	+
44	WP_004718988.1	Hypothetical 16	Endonuclease/Exonuclease	56,471	+	-	+	-	-	-	-	+	-
45	WP_004723305.1	LpoA/LppC[a]	Peptidoglycan synthesis	74,138	+	-	-	+	-	-	-	-	+
46	WP_004721574.1	LptD[a]	LPS assembly	89,597	+	-	-	-	-	-	-	-	+
47	WP_004721563.1	Hypothetical 20[a]	Membrane associated	17,747	+	-	-	-	-	-	-	-	-
48	WP_004722010.1	FhuA[a]	Ferric hydroxymate receptor	81,661	+	-	-	-	-	-	-	-	+
49	WP_004723308.1	YraP	Phospholipid biogenesis	20,176	-	+	+	+	+	+	-	+	+
50	WP_004719577.1	Pep M37[a]	Enzymatic activity	50,517	-	+	-	-	-	-	-	-	+
51	WP_004717303.1	Lipoprotein 1[a]	Unknown	20,214	-	+	-	-	-	-	-	-	+
52	WP_004722456.1	Blc[a]	Lipid storage/transport	20,700	-	+	-	-	-	-	-	-	+
53	WP_004720682.1	FliD	Flagellar filament cap	49,084	-	-	+	-	+	+	-	-	+
54	WP_004719093.1	Hypothetical 18[a]	Unknown	54,586	-	-	+	+	-	-	-	-	-
55	WP_004718688.1	YajG[a]	Unknown	20,929	-	-	+	-	-	-	-	-	+
56	WP_004719967.1	OmpW[a]	Hydrophobic compound transport	23,880	-	-	+	-	-	-	-	-	+
57	WP_004719291.1	MltC[a]	Peptidoglycan maintenance	39,902	-	-	+	-	-	-	-	-	+

Table S-2 Continued

	NCBI RefSeq number	Protein	Function	MW (Da)	Rainbow trout				Atlantic salmon				Prediction
					RD6	RD28	RD64	RD124	RD354	RD366	RD382	RD420	
58	WP_004718346.1	Hypothetical 19[a]	Unknown	51,997	-	-	+	-	-	-	-	-	-
59	WP_004722010.1	RupA[b]	Ruckerbactin reception	17,892	-	-	-	-	-	+	-	-	-
60	WP_004722673.1	SecD[a]	General secretion path.	71,754	-	-	-	+	-	-	-	-	+
61	WP_004717910.1	FadL[b]	Fatty acid compound transport	46,063	-	-	-	-	+	-	-	+	+
62	WP_004721213.1	TpsB family[b]	Hemolysin activator	61,725	-	-	-	-	-	-	+	+	-
63	WP_004720181.1	RlpA[b]	Rod shape cell seperation	37,557	-	-	-	-	-	-	-	+	+
64	WP_004718110.1	Phospholipase A1[b]	Bacteriocin secretion	32,533	-	-	-	-	-	-	-	+	+
				**Total**	**46**	**40**	**40**	**40**	**37**	**38**	**33**	**37**	**48**

Boxes coloured red, orange and yellow represent proteins identified in 3, 2 and 1 replicate, respectively. [a]Proteins identified solely in isolates recovered from Rainbow trout; [b]Proteins identified solely in isolates recovered from Atlantic salmon.

Table S-3 Additional OMPs identified through gel-based proteomics of *Y. ruckeri*.

	NCBI RefSeq number	Protein	Function	MW (Da)	Gel position[a]	Prediction
1	WP_004718264.1	YiaD/Omp16	Multicopy suppressor of BamD	22,248	20	+
2	WP_004720181.1	RlpA	Rod shape cell separation	37,557	38	+
3	WP_042527118.1	Surface Ag	Antigen	64,555	41	-
4	WP_038241510.1	MalA	Macrolide transporter	40,203	42	-
				**Total**	**4**	**2**

[a]These numbers represent the position of the protein on the gel in Fig. S-2

Table S-4 **Proteins identified in eight isolates of *Y. ruckeri* through a combination of gel-free and gel-based proteomic techniques.**

	NCBI RefSeq number	Protein	Function	MW (Da)	Predicted	Gel-free	L-S[a]	I-B[b]	Other-gel
1	WP_071704305.1	OmpF	Porin (Small molecules)	40,049	+	+	+	+	-
2	WP_004700255.1	Lpp (Brauns)	Anchors peptidoglycan to OM	6,687	+	+	+	+	-
3	WP_004717101.1	OmpE	Inorganic phosphate uptake	39,949	+	+	+	+	-
4	WP_004717609.1	Pcp/SlyB	Membrane integrity	15,334	+	+	+	+	-
5	WP_004717812.1	OmpC.1	Porin (Small molecules)	40,484	+	+	+	+	-
6	WP_004717910.1	FadL	Fatty acid compound trans.	46,063	+	+	+	+	-
7	WP_004717911.1	VacJ/MlaA	Phospholipid homeostasis	28,735	+	+	+	+	-
8	WP_004718110.1	Phospholipase A1	Bacteriocin secretion	32,533	+	+	+	+	-
9	WP_004718688.1	YajG	Unknown	20,929	+	+	+	+	-
10	WP_004718926.1	OmpA	OM integrity, porin, adherence	37,756	+	+	+	+	-
11	WP_004719473.1	BtuB	Vitamin B12 transport	68,999	+	+	+	+	-
12	WP_004719617.1	MetQ	D-methionine binding	29,316	+	+	+	+	-
13	WP_004719660.1	BamA/YaeT	BAM complex	88,077	+	+	+	+	-
14	WP_004719859.1	MipA	MltA interacting	28,040	+	+	+	+	-
15	WP_004719967.1	OmpW	Hydrophobic compound transport	23,880	+	+	+	+	-
16	WP_004720193.1	LptE/RlpB	LPS assembly	21,089	+	+	+	+	-
17	WP_004720628.1	OmpC.2	Porin (Small molecules)	39,971	+	+	+	+	-
18	WP_004720730.1	FlgE	Links flagella to driver	44,281	+	+	+	+	-
19	WP_004720881.1	ShuA	Heme transport	73,397	+	+	+	+	-
20	WP_004721031.1	TolC	OM Channel (Efflux system)	53,080	+	+	+	+	-
21	WP_004721574.1	LptD	LPS assembly	89,597	+	+	+	+	-
22	WP_004721707.1	CsgG	Curli organelles/biofilm formation	24,670	+	+	+	+	-

Table S-4 Continued

	NCBI RefSeq number	Protein	Function	MW (Da)	Predicted	Gel-free	L-S[a]	I-B[b]	Other-gel
23	WP_004722151.1	Pal	Peptidoglycan associated	18,103	+	+	+	+	-
24	WP_004722316.1	BamC	BAM complex	38,311	+	+	+	+	-
25	WP_004723020.1	TamA/YtfN	Autotransporter assembly	63,376	+	+	+	+	-
26	WP_004723105.1	BamD	BAM complex	27,392	+	+	+	+	-
27	WP_004723240.1	BcsC	Cellulose biosynthesis	126,490	+	+	+	+	-
28	WP_038251462.1	Flagellin (FlaA)	Flagellin	43,615	+	+	+	+	-
29	WP_004717303.1	Lipoprotein 1	Unknown	20,214	+	+	+	-	-
30	WP_004717998.1	BamB	BAM complex	42,328	+	+	+	-	-
31	WP_004718497.1	OprC	Chitoporin	75,008	+	+	+	-	-
32	WP_004719715.1	MltA	Peptidoglycan maintenance	42,866	+	+	+	-	-
33	WP_004719814.1	Slp family	Structural and OM stabilisation	20,645	+	+	+	-	-
34	WP_004718264.1	YiaD/Omp16	Multicopy suppressor of BamD	22,248	+	+	-	-	+
35	WP_004720181.1	RlpA[d]	Rod shape cell separation	37,557	+	+	-	-	+
36	WP_004718917.1	PqiB/MAM7	Adherence	59,670	+	+	-	-	-
37	WP_004719291.1	MltC[c]	Peptidoglycan maintenance	39,902	+	+	-	-	-
38	WP_004719577.1	Pep M37[c]	Enzymatic activity	50,517	+	+	-	-	-
39	WP_004720126.1	Hypothetical 5	Unknown	31,358	+	+	-	-	-
40	WP_004720682.1	FliD	Flagellar filament cap	49,084	+	+	-	-	-
41	WP_004722010.1	FhuA	Ferric hydroxymate receptor	81,661	+	+	-	-	-
42	WP_004722118.1	TTSS protein	Type III secretion protein	61,640	+	+	-	-	-
43	WP_004722456.1	Blc	Lipid storage/transport	20,700	+	+	-	-	-
44	WP_004722544.1	ShlB/FhaC/HecB family	Hemolysin activator	62,461	+	+	-	-	-

Table S-4 Continued

	NCBI RefSeq number	Protein	Function	MW (Da)	Predicted	Gel-free	L-S[a]	I-B[b]	Other-gel
45	WP_004722673.1	SecD	General secretion path.	71,754	+	+	-	-	-
46	WP_004723305.1	LpoA/LppC	Peptidoglycan synthesis	74,138	+	+	-	-	-
47	WP_004723308.1	YraP	Phospholipid biogenesis	20,176	+	+	-	-	-
48	WP_004723375.1	ShlA/FhaA/HecA family	Adhesin	155,563	+	+	-	-	-
49	WP_038244358.1	OmpX	Virulence	17,086	+	-	+	+	-
50	WP_004718684.1	Tsx	Nucleoside channel/Colicin rec.	29,122	+	-	+	+	-
51	WP_099511713.1	PilV	Major Pilin	48,727	+	-	+	+	-
52	WP_042526217.1	Hypothetical 12	Unknown	11,794	+	-	+	+	-
53	WP_004720405.1	NlpI	Cell division	33,918	+	-	+	-	-
54	WP_038242024.1	FlgH	Flagella L-ring protein	13,149	+	-	+	-	-
55	WP_004717693.1	NlpC/CutF	Copper homeostasis	25,530	+	-	+	-	-
56	WP_038241300.1	BamE	BAM complex	12,651	+	-	+	-	-
57	WP_042524944.1	YdgA	GTP-binding protein	54,395	+	-	+	-	-
58	WP_004719021.1	LpoB	Peptidoglycan synthesis	20,698	+	-	-	+	-
59	WP_004722810.1	Hypothetical 13	Unknown	34,471	+	-	-	+	-
60	WP_038241996.1	FliF	Flagellar machinary	61,743	+	-	-	+	-
61	WP_004719968.1	OsmY	Osmoregulation	11,017	-	+	+	-	-
62	WP_004722010.1	RupA	Ruckerbactin reception	17,892	-	+	-	-	-
63	WP_004717487.1	Hypothetical 15	Unknown	34,982	-	+	-	-	-
64	WP_004717528.1	HslJ	Heat-inducible protein	15,597	-	+	-	-	-
65	WP_004717681.1	HemR	Iron transport	72,952	-	+	-	-	-
66	WP_004717779.1	TcyP	Cysteine transporter	48,096	-	+	-	-	-

Table S-4 Continued

	NCBI RefSeq number	Protein	Function	MW (Da)	Predicted	Gel-free	L-S[a]	I-B[b]	Other-gel
67	WP_004718068.1	YfhG/QseG	Quorum sensing/Virulence	34,809	-	+	-	-	-
68	WP_004718346.1	Hypothetical 19	Unknown	51,997	-	+	-	-	-
69	WP_004718988.1	Hypothetical 16	Unknown	56,471	-	+	-	-	-
70	WP_004719093.1	Hypothetical 18	Unknown	54,586	-	+	-	-	-
71	WP_004721213.1	TpsB family	Hemolysin activator	61,725	-	+	-	-	-
72	WP_004722195.1	YqjD	Unknown	26,277	-	+	-	-	-
73	WP_004722541.1	Filamentous hemagglutinin, partial	Filamentous haemaglutinnin	48,764	-	+	-	-	-
74	WP_004723357.1	C-terminal protease	Unknown	75,933	-	+	-	-	-
75	WP_004723390.1	YeeJ	Unknown	54,668	-	+	-	-	-
76	WP_004717668.1	GlpC	Iron-Sulfur protein	115,110	-	-	+	+	-
77	WP_004721563.1	Hypothetical 20	Unknown	17,747	-	+	+	+	-
78	WP_004723092.1	Membrane protein	Unknown	27,916	-	-	+	+	-
79	WP_038242171.1	Lipoprotein 3	OM lipoprotein	11,336	-	-	+	-	-
80	WP_004719153.1	NlpD	Unknown	34,142	-	-	-	+	-
81	WP_004717948.1	YegD/Hsp70	Heat shock protein	49,742	-	-	-	+	-
82	WP_004721184.1	DauA	Sodium-independent anion transport	60,560	-	-	-	+	-
83	WP_038241510.1	MalA	Macrolide transporter	40,203	-	-	-	-	+
84	WP_042527118.1	Surface Ag	Antigen	64,555	-	-	-	-	+
				**Total**	**60**	**64**	**47**	**41**	**4**

[a]L-S represents the lane-section approach; [b]I-B represents the individual band approach; [c]Proteins identified solely in isolates recovered from Rainbow trout; [d]Proteins identified solely in isolates recovered from Atlantic salmon.

Table S-5 Functional categories of 141 predicted OMPs in four *Y. ruckeri* genomes

al Category	Total number of proteins	Number of core proteins (%)
OM Biogenesis and integrity	15	12 (80)
Transport and receptor	29	15 (52)
Adherence	16	4 (25)
Enzymatic activities	19	8 (42)
Motility	10	3 (30)
Other function	21	3 (12)
Unknown function	17	2 (14)
Hypothetical	14	1 (7)
